# Customized Sized Manganese Sulfide Nanospheres as Efficient T_1_ MRI Contrast Agents for Enhanced Tumor Theranostics

**DOI:** 10.34133/bmr.0116

**Published:** 2024-12-11

**Authors:** Yufang Gong, Kai Guo, Siyu Cai, Ke Ren, Liya Tian, Yingqi Wang, Mengyao Mu, Qingwei Meng, Jie Liu, Xiao Sun

**Affiliations:** ^1^ Department of Medical Oncology, Harbin Medical University Cancer Hospital, Harbin 150081, Heilongjiang, China.; ^2^ Department of Radiation Oncology & Shandong Provincial Key Laboratory of Radiation Oncology, Shandong Cancer Hospital and Institute, Shandong First Medical University & Shandong Academy of Medical Sciences, Jinan 250000, China.

## Abstract

The impact of nanoparticle size on the effectiveness of magnetic resonance imaging (MRI) using sulfurized manganese nanoparticles (MnS@PAA) stabilized with polyacrylic acid (PAA) as a binder was thoroughly investigated. MnS@PAA nanoparticles of varying sizes were synthesized by altering the ratio of ethylene glycol (EG) to diethylene glycol (DEG) during the synthesis process. These nanoparticles exhibited a uniform size distribution and demonstrated high T_1_ relaxation rates, along with a notable pH-responsive behavior. As the nanoparticle size increased, the T_1_ relaxation rate decreased, indicating that size plays a crucial role in their MRI performance. Additionally, research has revealed that the efficiency of tumor uptake by these nanoparticles is size dependent. Specifically, MnS@PAA nanoparticles with a core size of 100 nm (MS_100_) exhibited greater tumor accumulation and provided enhanced MRI contrast. Once within the acidic environment of a tumor, MS_100_ decomposes into Mn^2+^ and H_2_S. Mn^2+^ ions promote the generation of hydroxyl radicals, which leads to lipid peroxidation and induces ferroptosis. Concurrently, the release of H_2_S inhibits catalase activity, resulting in elevated levels of hydrogen peroxide (H_2_O_2_), achieving a synergistic effect between chemodynamic therapy (CDT) and gas therapy. This study explores the influence of nanoparticle size on its potential applications as an MRI contrast agent and as a therapeutic agent in cancer treatment.

## Introduction

Magnetic resonance imaging (MRI) is currently one of the most powerful tools in medical diagnostics due to its capacity to capture 3-dimensional (3D) tomographic information of entire tissues with high spatial and temporal resolution [1–5]. Unlike computed tomography (CT) and positron emission tomography (PET)/CT, MRI does not involve any radionuclides, making it more suitable for repeated examinations. However, MRI contrast relies on signal differences between tissues, and these inherent variations are often insufficient to clearly distinguish pathological from normal tissues. Therefore, MRI contrast agents are necessary to enhance image contrast and improve diagnostic accuracy. Enhancing the performance of nanoparticle (NP)-based MRI contrast agents can be achieved through surface modification and by optimizing their intrinsic physicochemical properties. Researchers have extensively explored improving the magnetic resonance performance of contrast agents by selecting magnetic materials, optimizing synthesis methods, conducting surface modifications, controlling the size and shape of NPs, and designing magnetic nanocomposites.

Gadolinium-based contrast agents are among the most commonly used in clinical diagnostics. Currently, gadopentetate dimeglumine is widely utilized as a T_1_ contrast agent in clinical MRI, particularly for brain tumor imaging. However, gadolinium-based contrast agents pose risks, including renal impairment, nephrogenic systemic fibrosis, and potential abnormal accumulation in the brain [6–8]. These risks underscore the need to develop new multifunctional contrast agents with lower toxicity for clinical use.

Manganese (Mn), with its 5 unpaired 3d electrons, is a potential MRI T_1_ contrast agent with lower toxicity than gadolinium, making it a promising alternative for contrast agent development. Currently, various Mn-based MRI contrast agents have been extensively studied in both oncology and non-oncology fields, including Mn sulfide (MnS) [9–11], Mn oxides [12–15], Mn carbonate [16,17], Mn ferrite [18–20], and other Mn-containing contrast agents [21–23]. Among these Mn-based MRI contrast agents, the γ-type MnS (γ-MnS) stands out due to its acid-responsive characteristics. Under acidic conditions, γ-MnS can decompose and produce 2 components: Mn^2+^ and H_2_S gas [9]. Mn^2+^ not only provides strong MRI T_1_ imaging performance but also converts H_2_O_2_ in tissues into toxic hydroxyl radicals via the Fenton-like reaction to eliminate tumor cells. H_2_S effectively inhibits intracellular catalase (CAT) activity, providing a continuous source of substrates for the Fenton-like reaction [24,25]. This dual-functional γ-MnS theranostic platform offers a novel approach for individualized detection and therapy of diseases [26].

Researchers are focused on studying the cellular uptake mechanisms of various nanomaterials, such as size and other factors, to enhance NP accumulation in diseased areas and improve diagnostic sensitivity. However, the complex biochemical environment of the human body results in varying biodistribution and cellular uptake of NPs of different sizes. Generally, NPs within the size range of 10 to 200 nm are suitable for cellular uptake, but determining the optimal size for imaging agents requires further research. Currently, studies on the impact of particle size on relaxation are primarily focused on iron oxide NPs [27,28] However, T_2_ contrast agents inherently suffer from drawbacks, including a lower signal-to-noise ratio (SNR), susceptibility to artifacts, and difficulties in distinguishing between different tissue types [29]. These limitations underscore the need for alternative contrast agents that can overcome these challenges while providing clearer and more accurate imaging results. Therefore, it is imperative to explore the synthesis of MnS NPs of varying sizes and assess their T_1_ imaging effectiveness in tumor environments.

The solvothermal method for synthesizing MnS nanomaterials offers unique advantages. The choice of solvent is critical for the final product's properties. The physicochemical properties of the solvent, such as viscosity, polarity, and surface tension, influence chelation efficiency as well as the nucleation and growth of NPs. For instance, solvents with higher viscosity can stabilize smaller NPs, preventing excessive growth and aggregation [30]. The polarity of the solvent influences the solubility and distribution of metal ions, thereby controlling the size and shape of NPs [31]. Chelation not only plays a role in controlling the nucleation rate and growth direction but also is crucial in enhancing the purity and quality of the final product. Thus, the solvothermal method is suitable for synthesizing nanomaterials with specific sizes and shapes and is vital for precisely controlling the material’s chemical composition and structure [32]. This method has broad application prospects in energy, environmental, and biomedical fields, particularly in the development of nanomaterials of various sizes.

**Fig. 1.**
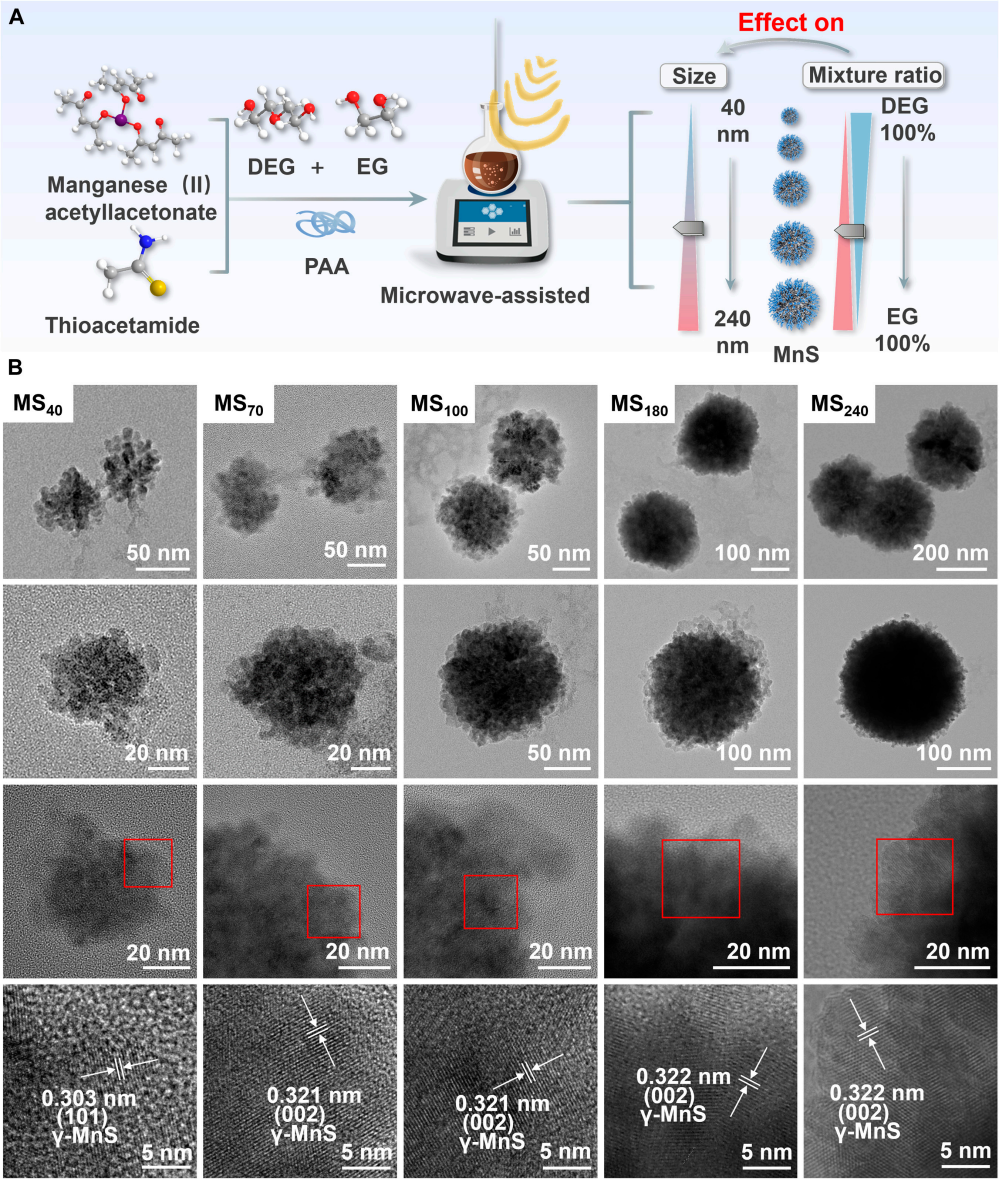
Preparation and morphology of MnS@PAA. (A) Schematic representation of the preparation of MnS@PAA NPs in different sizes. (B) TEM and high-resolution TEM images showing the morphology of MnS@PAA NPs with varying sizes.

In this study, we successfully synthesized 5 spherical γ-MnS (MnS@PAA) particles with distinct sizes using the solvothermal method and by regulating the proportion of solvent incorporation [30,32] (Fig. 1A). We explored their cellular uptake capability and imaging proficiency, ultimately selecting the variant with the superior imaging effect to investigate its efficacy in tumor therapy and underlying mechanisms. The results demonstrated that the 5 synthesized MnS@PAA particles were efficiently internalized by cells and accumulated at the tumor site during in vivo imaging, showing size-dependent differences in uptake and imaging due to the enhanced permeability and retention effect. Mechanistically, the synthesized MnS@PAA rapidly decomposes in the acidic environment, allowing for gradual diffusion of Mn^2+^, which marked enhances MRI capability at the tumor site. Simultaneously, the produced hydrogen sulfide can effectively inhibit CAT activity [33], resulting in an increased deposition of H_2_O_2_ at the tumor site. Moreover, this continuous generation of H_2_O_2_ provides ample substrate for the Mn^2+^-mediated Fenton reaction, amplifying ferroptosis in tumor cells through a cascade effect [34,35]. In summary, this study presents an innovative approach for the widespread application of MnS@PAA in nanomaterial carriers.

## Materials and Methods

### Materials

Acetylacetone Mn was purchased from Bide Pharmatech Co. Ltd. (Shanghai, China). Thioacetamide, sodium sulfide, fluorescein isothiocyanate (FITC), Cy5.5, and methylene blue (MB) were purchased from Shanghai Aladdin Biochemical Technology Co. Ltd. (Shanghai, China). Ethanol, ethylene glycol (EG), and diethylene glycol (DEG) were obtained from Sinopharm Chemical Reagent Co. Ltd. (Shanghai, China). 3-(4,5-Dimethylthiazol-2-yl)-2,5-diphenyltetrazolium bromide (MTT) was acquired from Sangon Biological Engineering Technology & Services Co. Ltd. (Shanghai, China). 2′,7′-Dichlorodihydrofluorescein diacetate (DCFH-DA) was sourced from MedChemExpress (USA). Calcein AM/propidium iodide (PI) dye was purchased from Beyotime Biotechnology (Shanghai, China). Hoechst 33342 was obtained from Sigma-Aldrich (USA). Membrane protein V-FITC and 7-AAD apoptosis detection kits were bought from Meilunbio Co. Ltd. (Dalian, China). GPX4 antibody and glyceraldehyde-3-phosphate dehydrogenase (GAPDH) antibody were sourced from Proteintech (USA). Fetal bovine serum (FBS) was purchased from Gibco (USA). Phosphate-buffered saline (PBS) and Dulbecco’s modified Eagle’s medium (DMEM) were obtained from Biological Industries Israel Beit-Haemek Ltd. (Kibbutz Beit-Haemek, Israel). Penicillin–streptomycin solution and recombinant trypsin–EDTA solution were also provided by Beyotime. All the reagents were used as received without further purification. Throughout the entire experiments, deionized water with a resistivity higher than 18 MΩ cm was used.

### Synthesis of MnS@PAA

The synthesis of MnS@PAA was conducted as follows: 213.174 mg (600 mM) of Mn acetylacetonate was dissolved in 20 ml of DEG. To this solution, 200 mg of polyacrylic acid (PAA) was added. The mixture was stirred at 350 rpm for 2 h at 75 °C. Afterward, 56.348 mg (750 mM) of thioacetamide was added to the reaction system and allowed to dissolve. Then, the resulting mixture was transferred to a microwave synthesizer, where the temperature was maintained at 200 °C for 1 h. The product was washed with anhydrous ethanol, centrifuged, and stored in anhydrous ethanol. At this stage, the particle size of the synthesized MnS was about 40 nm. For the synthesis of MnS with 4 other sizes, the solvents were modified to 18 ml of DEG + 2 ml of EG (~70 nm), 16 ml of DEG + 4 ml of EG (~100 nm), 12 ml of DEG + 8 ml of EG (~180 nm), and 20 ml of EG (~240 nm) while keeping all other synthesis details the same.

### Characterization of MnS@PAA

Transmission electron microscopy (TEM; Talos F200i, Thermo Fisher Scientific, MA, USA) was used to observe the microstructure and morphology of MnS@PAA. Particle size and zeta potential were analyzed using dynamic light scattering (DLS; Malvern Zetasizer Nano ZS, UK). Fourier transform infrared spectroscopy (FT-IR; Thermo Fisher Scientific Nicolet iS5, USA) and x-ray diffraction (XRD; BRUKER D8 AVANCE, Germany) were utilized to characterize the structure of MnS@PAA. The influence of MnS@PAA on the ultraviolet–visible (UV–vis) spectrum of MB was assessed using a UV–vis spectrophotometer (Genesys 50, USA). Cellular uptake fluorescence intensity was monitored using an inverted fluorescence microscope (Leica DMi8, Germany). Metal ion concentrations in the nanomaterials were quantified using inductively coupled plasma optical emission spectroscopy (ICP-OES; Agilent 5800 VDV, USA). The biodistribution of MnS@PAA in mice was tracked using a small-animal in vivo optical 3D imaging system (PerkinElmer IVIS Spectrum CT, USA). Vibration sample magnetometry (VSM, LakeShore7404, USA) is used to measure the paramagnetism of MnS@PAA. T_1_-weighted MRI in vitro and in vivo was performed using a 1.5-T scanner (HT-MRSI60-35A, Shanghai), a 3.0-T clinical scanner (Siemens MAGNETOM Prisma, Germany), and a 9.4-T animal scanner (Bruker BioSpec 94/30, Germany).

### The release of Mn^2**+**^ triggered by acidic environment

The release of Mn^2+^ triggered by an acidic environment was analyzed quantitatively using ICP-OES. Initially, equal amounts of MnS@PAA (5 mg) were dispersed into PBS solutions with pH 5.5 and 7.4, and then incubated at room temperature for 24 h. Samples were collected at intervals of 0, 0.5, 1, 2, 4, 8, 12, and 24 h. After centrifugation, the supernatants were retained and analyzed with ICP-OES to determine the concentration of Mn^2+^ released at each time point.

### Hydroxyl radical generation capacity assessment

#### pH-dependent MB trends

In 2 ml of PBS at varying pH levels (5.5, 6.5, and 7.4), sodium bicarbonate (NaHCO_3_) was added to reach a final concentration of 25 mM. Then, 5 μl of 30% hydrogen peroxide (H_2_O_2_) and MB were added to reach a concentration of 10 μg/ml. After thorough mixing, 0.5 mM (Mn^2+^) MS_100_ was introduced. Absorbance was measured every 30 s using a UV–vis spectrophotometer to generate pH-dependent MB absorption curves. Further measurements were conducted at 60, 90, and 120 s across all pH levels (5.5, 6.5, and 7.4) to document the changes in MB absorption over time.

#### Concentration-dependent MB trends

In 2 ml of PBS (pH 5.5), NaHCO_3_ was added to achieve a final concentration of 25 mM. Subsequently, 5 μl of 30% H_2_O_2_ and MB were added to reach a concentration of 10 μg/ml. After thorough mixing, 0, 0.25, and 0.5 mM of MS_100_ were introduced. Absorbance was measured every 30 s using a UV–vis spectrophotometer to conclude a concentration-dependent MB absorption curve. Measurements were continued at 60, 90, and 120 s to track the temporal changes in MB absorption depending on the concentration.

### MRI experiment

Concentrations of MnS@PAA and Magnevist at 0.03125, 0.0625, 0.125, 0.25, and 0.5 mM (Mn^2+^) were prepared in PBS at pH 5.5 and 7.4, respectively. After incubating for 10 min, the solutions were centrifuged, and the supernatants were analyzed using both a 0.5-T MRI scanner and a 3.0-T MRI scanner to evaluate the relaxation efficiency of the 5 MnS@PAA formulations. Then, Lewis lung carcinoma (LLC) cells (1 × 10^7^ cells/ml) were suspended in 1 ml of serum-free medium, and 100 μl was injected subcutaneously into the right side of C57BL/6 mice to establish a tumor model for in vivo MRI.

MnS@PAA of different sizes was incubated with LLC cells (3 × 10^5^ cells/dish) for 3 h. After incubation, the supernatant was removed, and the cell pellets were resuspended in PBS, embedded in 1% low-melting agarose gel, and analyzed for the relaxation efficiency of MnS@PAA in cells using a 9.4-T MRI scanner.

MnS@PAA was administered intravenously at 2 mg (Mn^2+^)/kg to these mice. MRI effects at different time points were evaluated using a 9.4-T MRI scanner with a maximum gradient strength of 660 mT/m and a slew rate of 4,570 T/m/s. The outer/inner diameter was 75 mm/40 mm. Scout images were taken in coronal and axial views before each session. T_1_-weighted imaging parameters included the following: echo time = 6 ms, repetition time = 800 ms, excitation angle = 90°, field of view = 4.0 × 3.0 cm^2^, matrix size = 256 × 256, slice thickness = 0.7 mm, and 17 repetitions per slice.

### Cell culture and biocompatibility/cytotoxicity assay

HUVEC, NIH 3T3, LX-2, and LLC cells were incubated at 37 °C and 5% CO_2_ in Dulbecco’s modified Eagle’s medium (DMEM) containing 10% FBS and 1% antibiotics. Subsequently, these cells were seeded onto a 96-well plate (approximately 1 × 10^4^ per well) and cultured overnight. MS_100_ at different concentrations was added to the wells. After 24 h of incubation, the medium was removed, followed by washing with PBS twice. One hundred microliters of 10% MTT solution (5 mg/ml) was then added to each well, and the plate was incubated for 3 h at 37 °C. The culture medium containing MTT was removed, and 150 μl of dimethyl sulfoxide (DMSO) was added to each well. The plate was shaken on a shaker for 15 min, and the absorbance at 490 nm was measured using a microplate reader.

### Cell uptake

Logarithmic growth phase LLC cells were seeded into 6-well plates (3 × 10^5^ cells/dish) and cultured for 24 h. Subsequently, cells were incubated with MnS@PAA of different sizes for 3 h [7.5 μg (Mn)/ml]. The cell pellets were treated with aqua regia to convert Mn into its ionic form and filtered through a 0.22-μm filter, and the Mn content was measured using ICP-OES.

Logarithmic growth phase LLC cells were seeded into coverglass-bottom dishes (3 × 10^5^ cells/dish) and cultured for 24 h. Subsequently, the first group was treated with MS_100_-FITC at different concentrations (0, 1.875, 3.75, and 7.5 μg/ml) for 4 h, followed by Hoechst 33342 staining for 30 min under dark conditions; the cells were then washed twice with PBS and observed using an inverted microscope. The second group was treated with 7.5 μg/ml MS_100_-FITC for varying durations (0, 1, 2, and 4 h), followed by Hoechst staining for 30 min under dark conditions; the cells were then washed twice with PBS and observed using an inverted microscope.

Following the same administration procedure as mentioned above, the cells were scraped off and the cell precipitate was collected and digested with aqua regia. The sample was then diluted with 2% nitric acid and filtered through a 0.22-μm filter, and the uptake capacity was determined using ICP-OES.

LLC cells were seeded in 6-well plates (3 × 10^5^ cells/well) and treated with MS_100_-FITC at different concentrations (0, 1.875, 3.75, and 7.5 μg/ml) for 4 h. The cells were washed twice with PBS and digested with trypsin without EDTA. Subsequently, flow cytometry (FCM) was employed for analysis.

Logarithmic growth phase LLC cells were seeded into coverglass-bottom dishes (3 × 10^5^ cells/dish) and cultured for 24 h. Afterward, the cells were treated in the following scenarios: (a) PBS, (b) MS_100_-FITC, (c) MS_100_-FITC at 4 °C, (d) MS_100_-FITC + amiloride, (e) MS_100_-FITC + chlorpromazine, and (f) MS_100_-FITC + methyl-β-cyclodextrin (MβCD). After a total incubation of 3.5 h, Hoechst staining was performed for 30 min under dark conditions; the cells were then washed twice with PBS and observed using an inverted microscope.

### Live/dead cell staining

The in vitro antitumor effect of MS_100_ was assessed using a cell viability/cytotoxicity assay kit that labels live cells with calcein AM (green fluorescence, excitation/emission = 494/517 nm) and dead cells with 7-aminoactinomycin D (7-AAD; red fluorescence, excitation/emission = 546/650 nm), respectively. In brief, LLC cells were seeded in 6-well plates (5 × 10^5^ cells/well) and cultured with MS_100_-FITC at concentrations of 0, 1.875, 3.75, and 7.5 μg/ml for 12 h. After 12 h, the cells were co-incubated with DMEM containing AM (2 μM) and PI (4.5 μM) under dark conditions for 30 min, and the staining results were observed using a fluorescence microscope.

### FCM detection of cell apoptosis

Apoptosis in LLC cells was assessed using the Annexin V-FITC apoptosis detection kit. LLC cells were seeded at 3 × 10^5^ cells/well in 6-well plates and exposed to MnS_100_ at final concentrations of 0, 1.875, 3.75, and 7.5 μg/ml, followed by incubation for 24 h. The cells were collected, washed twice with precooled PBS, and resuspended in 100 μl of binding buffer. Then, 5 μl of Annexin V-FITC and 5 μl of 7-AAD were added, and the mixture was incubated for 20 min at 37 °C in the dark. After dual staining, a total of 20,000 cells per sample were counted and analyzed using the Attune NxT Acoustic Focusing Cytometer.

### Hemolysis test

Mouse blood was washed to obtain red blood cells (RBCs), which were then resuspended in 20 ml of PBS. MS_100_ was added to the erythrocyte suspension at final concentrations of 3.125, 6.25, 12.5, 25, 50, 100, and 200 μg/ml. As a negative control, erythrocyte suspensions were diluted with PBS, while the suspensions with added ultrapure water served as the positive control. The suspensions were incubated at 37 °C for 3 h and then centrifuged, and photos were taken. The hemolysis rate was subsequently determined and calculated.

### Western blot assay

Cells were treated with different concentrations of MS_100_ (0 μg/ml, 3.75 μg/ml, 7.5 μg/ml, 7.5 μg/ml + 1 μM Fer-1) for 24 h. The cells were scraped off with a cell scraper and lysed, and the protein concentration was quantified using a BCA protein assay kit. Subsequently, the proteins were separated by 12% sodium dodecyl sulfate–polyacrylamide gel electrophoresis and transferred onto a polyvinylidene difluoride membrane (0.45 μm) for imaging. After blocking with 5% nonfat milk, the membrane was incubated sequentially with antibodies against GPX4 and GAPDH (1:1,000), followed by a horseradish peroxidase-conjugated goat anti-rabbit secondary antibody (1:5,000). Finally, the bands were visualized using an enhanced chemiluminescence detection kit.

### Intracellular H_2_S detection

LLCs were cultured on coverglass-bottom dishes for 24 h to allow cell attachment. The cells were then treated with MS_100_ at concentrations of 0, 1.875, 3.75, and 7.5 μg/ml for 24 h. A 15 μM H_2_S fluorescent probe WSP-1 was added for 30 min at 37 °C in the dark. After washing with PBS, the cells were observed under a fluorescence microscope (excitation/emission: 465/515 nm), and the fluorescence intensity was measured using ImageJ software.

### In vitro ROS assay

Reactive oxygen species (ROS) generation was monitored by confocal laser scanning microscopy (CLSM) using DCFH-DA fluorescent probe. Briefly, LLC cells were incubated with MS_100_ at concentrations of 0, 1.875, 3.75, and 7.5 μg/ml for 4 h. The cells were stained with DCFH-DA (10 μM) and Hoechst, incubated for 25 min, and washed with PBS twice. The fluorescence intensities of DCFH-DA were measured using a CLSM, and the fluorescence intensity was analyzed by ImageJ software.

### LPO and mitochondrial membrane potential evaluation

LLC cells were seeded in a confocal dish (1 × 10^5^ cells/dish) and cultured for 24 h. Subsequently, the cells were incubated with MS_100_ at concentrations of 0, 1.875, 3.75, and 7.5 μg/ml for 4 h. The cells were then stained with a JC-1 assay kit following the manufacturer's protocols. Lipid peroxidation (LPO) levels and mitochondrial membrane potential were subsequently visualized and analyzed using fluorescence microscopy.

### CAT activity assay

The impact of MS_100_ on the activity of CAT in cells was assessed using a hydrogen peroxide enzyme assay kit. Cells were initially cultured in a 6-well plate at a density of 50,000 cells/well for 24 h. The cells were then treated with MS_100_ at concentrations of 1.875, 3.75, and 7.5 μg/ml for 12 h, and the control group was treated with PBS for 12 h. After cell collection by scraping, cells were centrifuged, and the proteins were extracted. Subsequent detection was performed following the hydrogen peroxide enzyme assay protocol. Additionally, cell suspensions (2 ml) were treated with Na_2_S solutions at concentrations of 0, 3.75, 7.5, 15, 30, and 60 μg/ml, while the control group was treated with PBS. After 30 min, cells were centrifuged and washed 3 times, and the cell lysis buffer was used to collect cell lysates for CAT activity detection. The relative enzyme activity was calculated as follows: absorbance of each group/absorbance of the control group.

### Cell uptake and mitochondrial observation

LLC cells were co-incubated with 7.5 μg/ml of MS_100_ for 24 h and then scraped and centrifuged to collect the cell pellet. The pellet was slowly treated with 2.5% glutaraldehyde fixative, precooled at 4 °C, along the tube wall. After embedding and sectioning, the cellular uptake of nanomaterials and the damage to mitochondria caused by ROS were observed using a TEM.

### Real-time biodistribution imaging

The LLC tumor model was established subcutaneously on the right flank of female C57BL/6 mice (1 × 10^6^ cells/mouse). MnS@PAA-Cy5.5 NPs were then injected intravenously. Biodistribution was monitored in real time using an IVIS imaging system (Thermo Fisher Scientific, USA).

### Biosafety assessment

After treatment, LLC tumor-bearing mice were sacrificed to collect major organs (heart, liver, spleen, lungs, and kidneys) and blood samples. The tissue samples were fixed in a 4% paraformaldehyde solution and stained with hematoxylin and eosin (H&E). Additionally, biochemical parameters in the blood were assessed.

### In vivo tumor suppression study

LLC cells were suspended in serum-free medium (1 × 10^7^ cells/ml), and 100 μl (1 × 10^6^ cells per mouse) was subcutaneously injected into the right flank of female C57BL/6 mice to establish a tumor-bearing model. Tumor volume (*V*) was calculated using the formula *V* = *a* * *b*^2^ * 0.52, where *a* and *b* represent the longest and shortest diameters of the tumor, respectively. When the tumors reached an initial size of approximately 100 mm^3^, the mice were randomly divided into 3 groups: PBS (G1), MS_100_ (G2), and MS_100_ + Fer-1 (G3), with 5 mice in each group. The mice were administered the drugs via intravenous injection in the tail vein, with a dosing regimen of MS_100_ at 2.0 mg (Mn)/kg (calculated based on the ICP results of Mn ions) every other day, and a Fer-1 dose of 10 mg/kg also every other day. Tumor volume and body weight were recorded every 2 days. The mice were euthanized on day 15 after treatment, and tumors were then extracted, weighed, and photographed.

### Statistical analysis

All data are expressed as the mean ± SD. *P* < 0.05 was considered to be statistically significant. Asterisks (*) denote statistical significance between bars (*P < 0.05, **P < 0.01, ***P < 0.001) conducted using one-way analysis of variance (ANOVA) and two-way ANOVA.

## Results

### Size-controlled synthesis and characterization of MnS@PAA

MnS NPs (MnS@PAA) were synthesized using a solvothermal method with PAA as a binder. The particle size of MnS@PAA was controlled by adjusting the ratio of DEG to EG. As the doping percentage of DEG/(DEG + EG) gradually increased from 0% to 100%, the size of MnS@PAA NPs observed through TEM also decreased accordingly. Under other fixed reaction conditions, the highest ratio of DEG/(DEG + EG) (100%) produced particles with the smallest size, approximately 40 nm. As the ratio of DEG/(DEG+EG) decreased to 90%, 80%, 60%, and 0%, the average particle size increased to 70, 100, 180, and 240 nm, respectively (Fig. 1B). For convenience, we labeled the samples as MS_40_, MS_70_, MS_100_, MS_180_, and MS_240_, respectively. It is important to note that MnS@PAA NPs exhibited continuous size tuning within the range of 40 to 240 nm while also maintaining good dispersibility and uniformity.

**Fig. 2.**
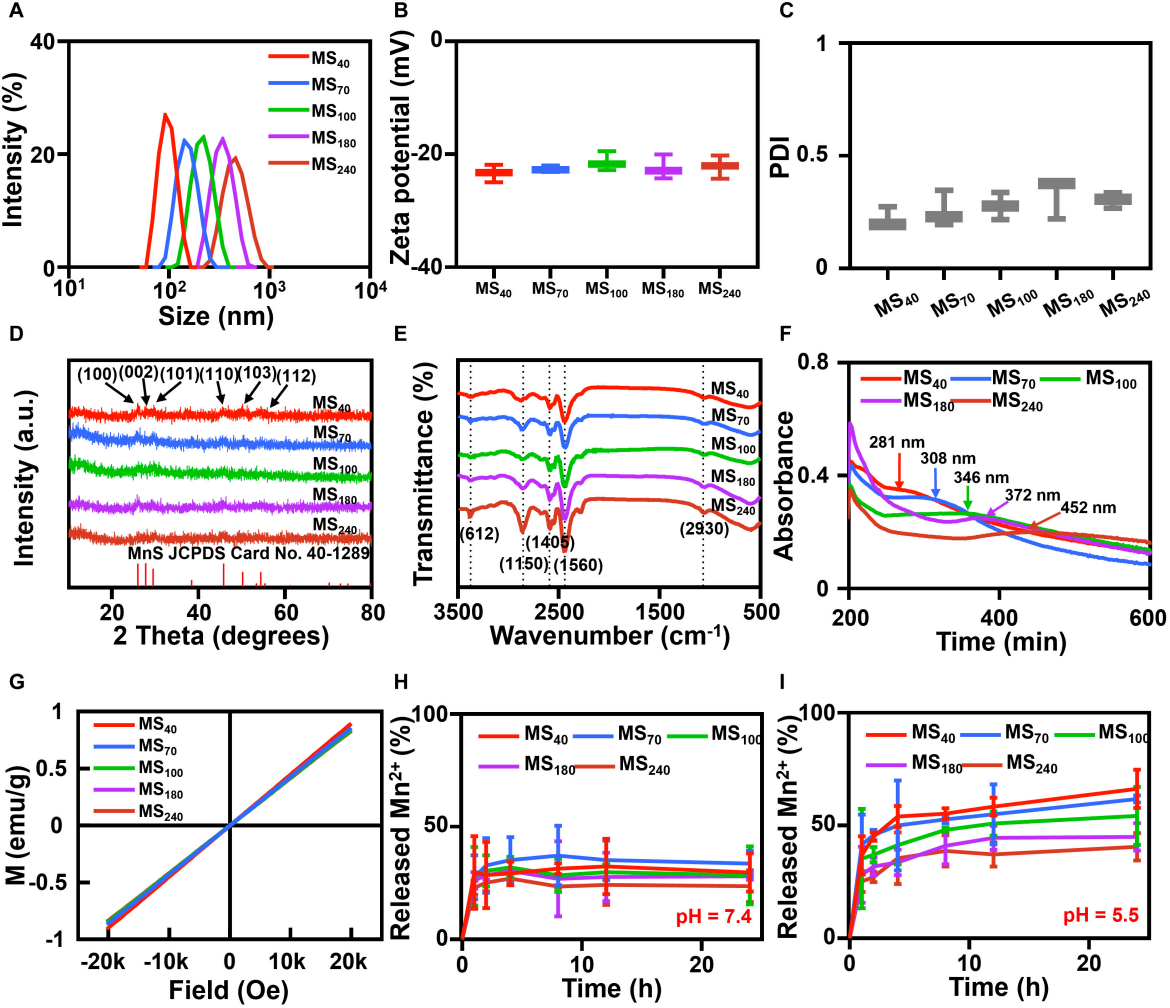
Characterization of MnS@PAA with different sizes. (A) DLS, (B) zeta potential, and (C) PDI of MnS@PAA with different sizes. (D) XRD and (E) FT-IR of MnS@PAA with different sizes. (F) Representative UV–vis spectra of MnS@PAA with different sizes. (G) Magnetic moment versus magnetic field plots of MnS@PAA with different sizes. (H) Release curves of Mn^2+^ from MnS@PAA with different sizes in pH 7.4 and (I) pH 5.5 in vitro (*n* = 3 independent experiments).

The hydrodynamic diameter of MnS@PAA NPs in solution was determined through DLS analysis (Fig. 2A). As the size of the MnS@PAA NPs increased from 40 nm to 70, 100, 180, and 240 nm, the observed hydrodynamic diameter also increased from 91.3 nm to 156.2, 223.5, 342.4, and 477.2 nm, respectively. As indicated by zeta potential measurements, all MnS@PAA NPs carried a highly negative charge, which helped to prolong the blood circulation time and stabilize the colloidal solution (Fig. 2B). Additionally, Fig. 2C shows that all 5 sizes of MnS NPs exhibited good dispersibility, with polydispersity index (PDI) values remaining below 0.5. Simultaneously, influenced by the quantum size effect, the absorption peak shifted from 281 to 452 nm as the size of the MnS nanospheres increased. This is because larger nanospheres have a smaller band gap, which reduces the required excitation energy [36], resulting in a redshift in the absorption spectrum to longer wavelengths (Fig. 2F).

XRD results showed that all 5 sizes of MnS@PAA have a similar structure, with 6 prominent peaks at 25.81°, 27.637°, 29.346°, 45.569°, 49.989°, and 54.127° corresponding to the (100), (002), (101), (110), (103), and (200) planes of γ-MnS (JCPDS No. 40-1289), confirming that the synthesized NPs across all sizes are γ-MnS (Fig. 2D). To further validate the successful synthesis of MnS@PAA NPs in different sizes, FT-IR analysis was performed on 5 varieties of MnS@PAA NPs. As expected, the 5 different sizes of MnS@PAA exhibited similar infrared spectra (Fig. 2E). Peaks between 506 and 612 cm^−1^ corresponded to the Mn–S stretching vibration in MnS. The characteristic peaks of PAA were observed at 1,560 and 1,405 cm^−1^, corresponding to the asymmetric and symmetric stretching vibrations of the carbonyl groups in acrylic acid. These results demonstrate the successful preparation of MnS@PAA NPs with different sizes, ranging from 40 to 240 nm, while maintaining consistent structural and dispersibility characteristics.

The VSM results present the room-temperature magnetic measurements of MnS@PAA samples with different particle sizes, under a magnetic field of up to 20 kOe (Fig. 2G). At 20 kOe, the magnetization values for the 5 samples are as follows: MS_40_, 0.89577 emu/g; MS_70_, 0.85624 emu/g; MS_100_, 0.83981 emu/g; MS_180_, 0.83549 emu/g; and MS_240_, 0.83177 emu/g. The results indicated that the magnetic-hysteresis curves of all MnS@PAA groups exhibited no hysteresis and displayed a linear relationship, suggesting that MnS@PAA of varying sizes possessed characteristics of paramagnetic materials.

### pH-responsive erosion of MnS@PAA

To investigate the acid responsiveness of γ-MnS, we studied the Mn ion release from MnS@PAA of varying sizes under different pH conditions (Fig. 2H and I). The results indicate a gradual increase in Mn ion detection in the solutions of all MnS@PAA samples over time. Furthermore, the release of Mn ions increased with decreasing pH values. Additionally, under pH 5.5 conditions, the smallest MnS@PAA particles exhibited the highest amount of Mn ion release (Fig. 2I and Table S1). These findings suggest that MnS@PAA displays acid responsiveness, with smaller NP sizes correlating with greater Mn ion release. This phenomenon may be attributed to the larger surface area of smaller NPs, facilitating increased proton interactions and consequently leading to enhanced Mn ion release.

### Size-dependent T_1_ relaxation performance of MnS@PAA

**Fig. 3.**
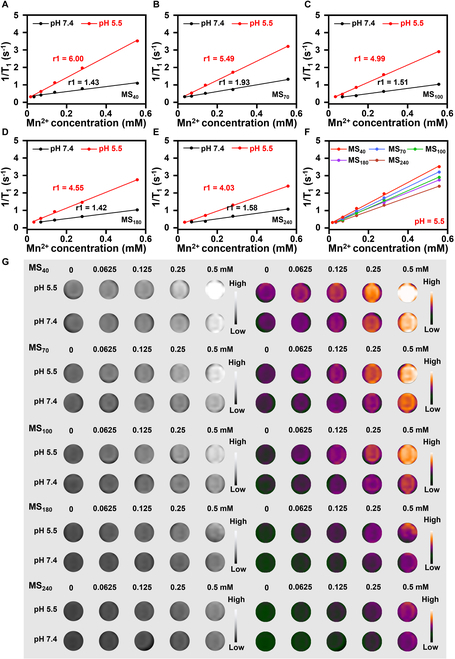
MRI performance of MnS@PAA with different sizes. (A to E) T_1_ relaxation rates of MnS@PAA with different sizes under various pH conditions from a 0.5-T MRI system in vitro. (F) T_1_ relaxation rates of MnS@PAA with different sizes in the same acidic environment (pH 5.5) in vitro. (G) T_1_-weighted MR image of MnS@PAA with different sizes from a 3.0-T MRI system.

The T_1_ relaxation performance of various sizes of MnS@PAA in different pH environments was then evaluated using a 0.5-T MRI scanner in vitro, aiming to explore the potential relationship between the efficiency of MRI contrast agents and the size of MnS@PAA (Fig. 3A to E). The results showed that at the same concentration of Mn^2+^, MnS@PAA with different sizes exhibited significantly higher longitudinal (r1) relaxivity values under acidic conditions (pH 5.5) compared to neutral conditions (pH 7.4), reaching 6.00, 5.49, 4.99, 4.55, and 4.03 mM^−1^ s^−1^, all of which were much higher than the r1 value of the current clinical contrast agent (Magnevist) at 2.88 mM^−1^ s^−1^ (Fig. 3F and Fig. S1). The 3.0-T MRI system also demonstrated a similar acid-responsive enhancement in relaxation (Fig. 3G and Fig. S1B). MnS@PAA samples of different sizes only exhibited minor differences in paramagnetism. Hence, the variations in the r1 values observed in MRI results are primarily attributed to the size-dependent acid responsiveness of the particles. Given that the tumor microenvironment is mildly acidic, this responsiveness provides γ-MnS with a potential advantage for use as a tumor MRI contrast agent. Thus, it is reasonable to consider MnS as a suitable candidate for MRI contrast agents in tumor environments.

### Size-dependent cellular uptake of MnS@PAA

**Fig. 4.**
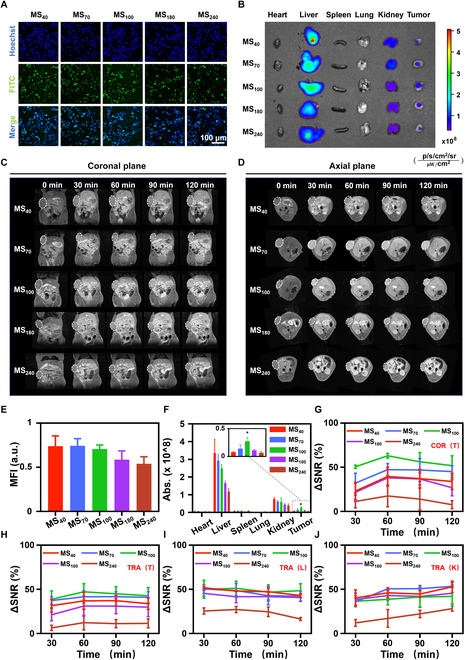
MRI performance and biodistribution of MnS@PAA with different sizes. (A) Size-dependent uptake capacity of MnS@PAA and (E) statistical analysis. Scale bar, 100 μm. (B) Ex vivo organ imaging of mice injected with different sizes of MnS@PAA and (F) statistical analysis. (C) Coronal plane of T_1_-weighted images of MnS@PAA in LLC-loaded mice in vivo and (G) its quantification. White dotted circles: tumor areas. (D) T_1_-weighted axis images of MnS@PAA in LLC-loaded mice in vivo and (H) its quantification. White dotted circles: tumor areas. (I) In vivo MR images of liver and (J) kidney at different time points. Data are represented as mean ± SD and analyzed by one-way ANOVA. *P < 0.05, ***P < 0.001 (*n* = 3 independent experiments).

To further investigate the uptake capacity of tumor cells for MnS@PAA of different sizes, FITC-labeled NPs were co-incubated with LLC cells, followed by qualitative and quantitative evaluations of the cellular uptake behavior. The results indicated an inverse relationship between the size of the NPs and their internalization by tumor cells. Specifically, smaller NPs exhibited greater cellular penetration and were more readily captured by cells (Fig. 4A and E). Subsequently, to further verify the cellular uptake efficiency of MnS@PAA NPs of different sizes, LLC cells co-incubated with MnS@PAA for 3 h were collected. The Mn content of different sizes taken up by the cells was measured using ICP-OES (Fig. S2). Additionally, MRI characterization was performed at the cellular level using samples embedded in 1% low-melting agarose gel (Fig. S3). The Mn content measured by ICP-OES and the MRI results of cell gels containing MnS@PAA NPs were consistent with the fluorescence results, all showing size-dependent cellular uptake.

### In vivo biocompatibility assessment of MnS@PAA

Before conducting in vivo MRI imaging research, we conducted an evaluation of the in vivo biocompatibility of MnS@PAA NPs of different sizes. First, MnS@PAA NPs of different sizes were injected into tumor-bearing mice to assess their blood biochemistry levels, followed by histopathological examination of key organs to comprehensively evaluate the biocompatibility of the nanomaterials. The results showed no significant hematological damage in the mice after treatment in each group (Fig. S4). H&E-stained liver sections from different mouse groups showed no apparent abnormalities or organ damage, confirming the absence of noticeable chronic toxicity (Fig. S5). Cardiac function parameters (creatinine kinase, lactate dehydrogenase) and renal function parameters (uric acid, blood urea nitrogen, and creatinine) remained within normal ranges, further demonstrating the overall safety of MnS@PAA NPs of varying sizes.

### In vivo MRI enhancement and biodistribution of MnS@PAA

To validate its MRI enhancement capability in vivo, a 2 mg (Mn)/kg dose of MnS@PAA with different sizes was intravenously injected into tumor-bearing mice via the tail vein. Compared with other groups, MS_100_ showed the strongest MRI enhancement ability at the same time point after injection, peaking at 60 min (ΔSNR = 62.56%), indicating that NPs of this size exhibit the most favorable in vivo MRI enhancement capacity (Fig. 4C, D , G, and H). The MRI performance at the tumor site of MnS@PAA with different sizes exhibited a trend that differed from the results of cellular uptake in tumor cells. Therefore, the variations in NP distribution were next explored through biodistribution studies (Fig. 4B and F). The results showed that Cy5.5-labeled MnS@PAA primarily accumulated in metabolic organs such as the liver and kidneys, as well as in tumors.

In addition to imaging at the tumor site, we compared the imaging of MnS@PAA NPs in the liver and kidneys. The results shown in Fig. S6 indicate that, except for the largest MS_240_, the other sizes of MnS@PAA exhibited similar metabolic trends: The signal in the liver diminished over time, while the MRI signal in the kidneys increased over time. Figure 3I and J provides a quantitative analysis of the signal changes in the liver and kidneys at different time points after the administration of the contrast agent, using ΔSNR as a metric.

In summary, the distribution of NPs revealed an unexpected pattern in tumors: MS_100_ demonstrated higher uptake, while other sizes of MnS@PAA were less prevalent. This discrepancy may be attributed to size-dependent cellular uptake and tissue penetration barriers. Larger NPs may face greater challenges in cellular internalization and tissue penetration, leading to reduced endocytosis efficiency. Conversely, smaller nanoparticles may be cleared more rapidly by the reticuloendothelial system, reducing their potential for tumor accumulation. The mid-sized MS_100_ appears to strike a balance, exhibiting both significant biodistribution and cellular uptake in tumors, resulting in relatively better imaging capabilities.

### Characterization and ROS production of MS_100_

**Fig. 5.**
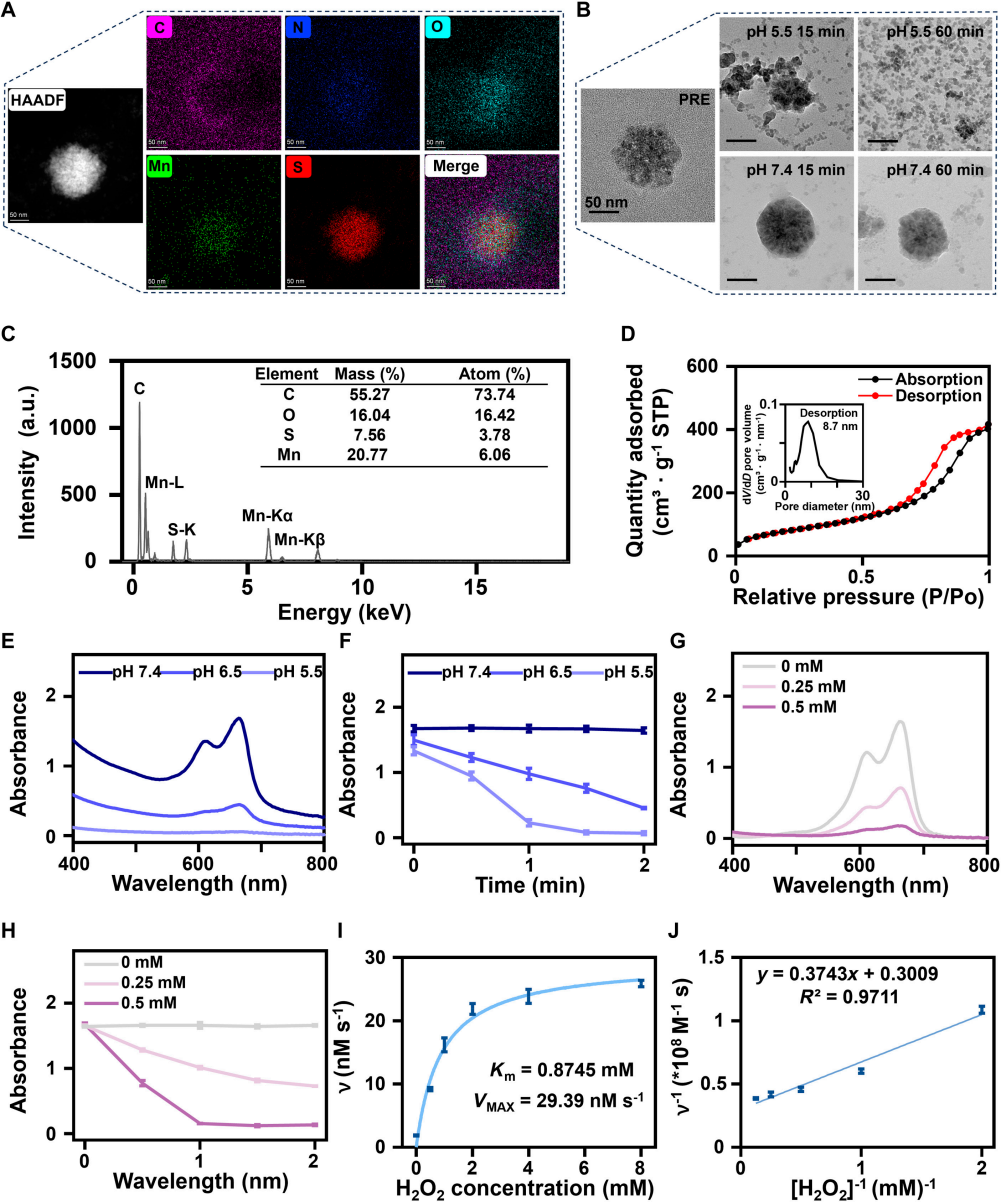
Characterization and properties of MS_100_. (A) High-angle annular dark-field (HAADF)–scanning TEM (STEM) images and elemental mapping of MS_100_. Scale bar, 50 nm. (B) TEM images of MS_100_ after incubation in different pH buffers (pH 5.5 and 7.4) for 15 and 60 min. Scale bar, 50 nm. (C) EDS spectrum of MS_100_. (D) N_2_ desorption isotherms and BJH desorption d*V*/d*D* pore volume of MS_100_. (E) Absorption spectra of MB at different pH levels. (F) Time-dependent trends in the absorption spectra of MB at different pH levels. (G) Absorption spectra of MB at different concentrations of MS_100_. (H) Time-dependent trends in the absorption spectra of MB at different concentrations of MS_100_. (I) Michaelis–Menten kinetics curve of MS_100_ related to TMB. (J) Double reciprocal plots of activity of MS_100_ with TMB as a substrate (*n* = 3 independent experiments).

Due to the outstanding MRI enhancement capabilities of MS_100_, we decided to conduct a detailed characterization of it. First, elemental mapping results confirmed the uniform distribution of Mn and S within the NPs (Fig. 5A). Meanwhile, energy-dispersive spectroscopy (EDS) analysis revealed that the MS_100_ NPs are composed of Mn, S, C, and O elements (Fig. 5C). Based on the rough surface of MnS observed in electron microscopy, we further assessed the drug-loading capacity of MS_100_ using Brunauer–Emmett–Teller measurements (BET measurements). The results showed that MS_100_ possesses a specific surface area of 292.35 m^2^/g, a pore volume of 0.65 cm^3^/g, and pore sizes determined to be between 7 and 9 nm by the Barrett–Joyner–Halenda method (BJH method), consistent with the spacing between MnS single crystals observed via TEM (Fig. 5D).

To further explore the pH-responsive degradation properties of MS_100_, we incubated it in buffer solutions with pH values of 5.5 and 7.4 for 15 and 60 min, respectively, and compared the TEM images before and after incubation. The results demonstrated that MnS rapidly degraded into single crystals around 10 nm in size under acidic conditions, with good dispersity (Fig. 5B). MB was used to detect the generation of hydroxyl radicals (•OH) by MS_100_ in the presence of H_2_O_2_. As shown in Fig. 5E and F, rapid production of •OH was observed within 2 min under acidic conditions at pH 5.5. In contrast, at pH 6.5, a relatively lower amount of •OH was produced, while in normal tissue conditions at pH 7.4, the production of •OH was negligible. These findings suggest that under acidic conditions, MS_100_ is more prone to releasing Mn^2+^, leading to the generation of significant amounts of •OH through Mn^2+^-mediated Fenton-like reactions, thereby demonstrating its potential for tumor cell eradication. Additionally, a concentration-dependent increase in ROS production was observed with increasing concentrations of MS_100_ (Fig. 5G and H). Finally, using TMB and H_2_O_2_ as substrates, the ROS production capability of MS_100_ via Mn^2+^ was further assessed through steady-state kinetics. Figure 5I illustrates the corresponding Michaelis–Menten model. The relationship between substrate concentration and reaction velocity was analyzed using a double reciprocal plot (Fig. 5J, Lineweaver–Burk plot). From this analysis, all reaction parameters were determined, yielding an equation *y* = 0.3743*x* + 0.3009 with an *R*^2^ value of 0.9711, *K*_m_ = 0.8745 mM, and *V*_max_ = 29.39 nmol/s.

### Cellular uptake, endocytosis pathways, and biocompatibility of MS_100_

**Fig. 6.**
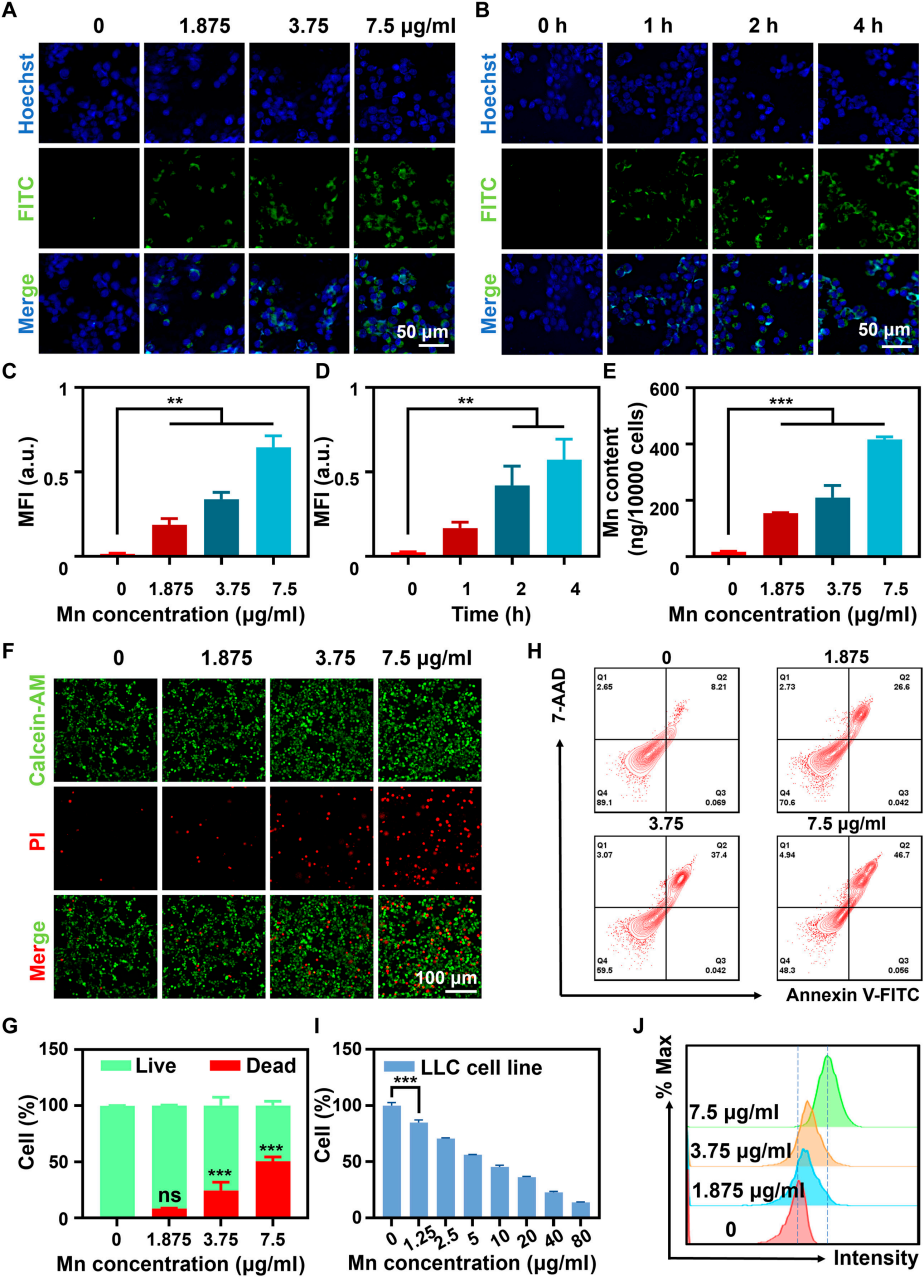
In vitro cellular uptake and antitumor activity of MS_100_. (A) Fluorescence images of LLC cells treated with varying doses of MS_100_ and (C) its quantification. (B) Fluorescence images of LLC cells treated with MS_100_ for 1, 2, and 4 h and (D) its quantification. (E) ICP-OES quantification of MS_100_ in LLC cells at increasing concentrations. (F) Calcein-AM/PI staining of LLC cells after MS_100_ treatment and (G) its relative quantification. (H) FCM analysis of cellular uptake. (I) Viability of LLC cells. (J) FCM analysis of apoptosis. Data are represented as mean ± SD and analyzed by one-way ANOVA. **P < 0.01, ***P < 0.001 (*n* = 3 independent experiments).

Fluorescence inverted microscopy results showed that LLC cells treated with MS_100_ exhibited dose- and time-dependent fluorescence signal enhancement, indicating a high uptake capacity for MS_100_ (Fig. 6A to D). This uptake ability was also confirmed by ICP-OES analysis and FCM (Fig. 6E and J). Subsequently, FITC-labeled MS_100_ NPs were co-incubated with cells in the presence of various endocytosis inhibitors to ascertain the internalization pathway of MS_100_ (Fig. S7). The results showed that fluorescence intensity within the cells was significantly reduced when experiments were conducted at 4 °C or when cells were pretreated with MβCD (a caveolae-dependent endocytosis inhibitor), indicating that uptake of the material was almost completely inhibited. Pretreatment with amiloride (a macropinocytosis inhibitor) had no effect on the uptake of NPs in LLC cells. However, pretreatment with chlorpromazine (a clathrin-mediated endocytosis inhibitor) partially inhibited the uptake. These findings demonstrate that the internalization of NPs in LLC cells is primarily driven by caveolae-mediated endocytosis, with some influence from clathrin-mediated endocytosis pathways (Fig. S8). These findings underscore the promising outlook of MnS@PAA with a particle size of 100 nm as the optimal nanodrug carrier.

Figure 6I shows the dependence of cell viability on the MS_100_ concentration. LLC cells were co-incubated with Mn chloride and sodium sulfide for 24 h, showing a concentration-dependent decrease in cell viability (Fig. S9). It is worth noting that MS_100_ exhibited very low cytotoxicity in normal cell lines such as HUVEC, NIH/3T3, and LX-2 cells, indicating its excellent biocompatibility (Fig. S10A). The hemolysis rate of MS_100_ was observed to be less than 5% in hemolysis tests, confirming its in vitro blood compatibility (Fig. S10B). Calcein-AM/PI staining and FCM analysis also indicated a similar degree of tumor cell death (Fig. 6F to H). These findings confirm that the synthesized MS_100_ exhibits excellent biocompatibility and effective tumor cell killing capabilities.

**Fig. 7.**
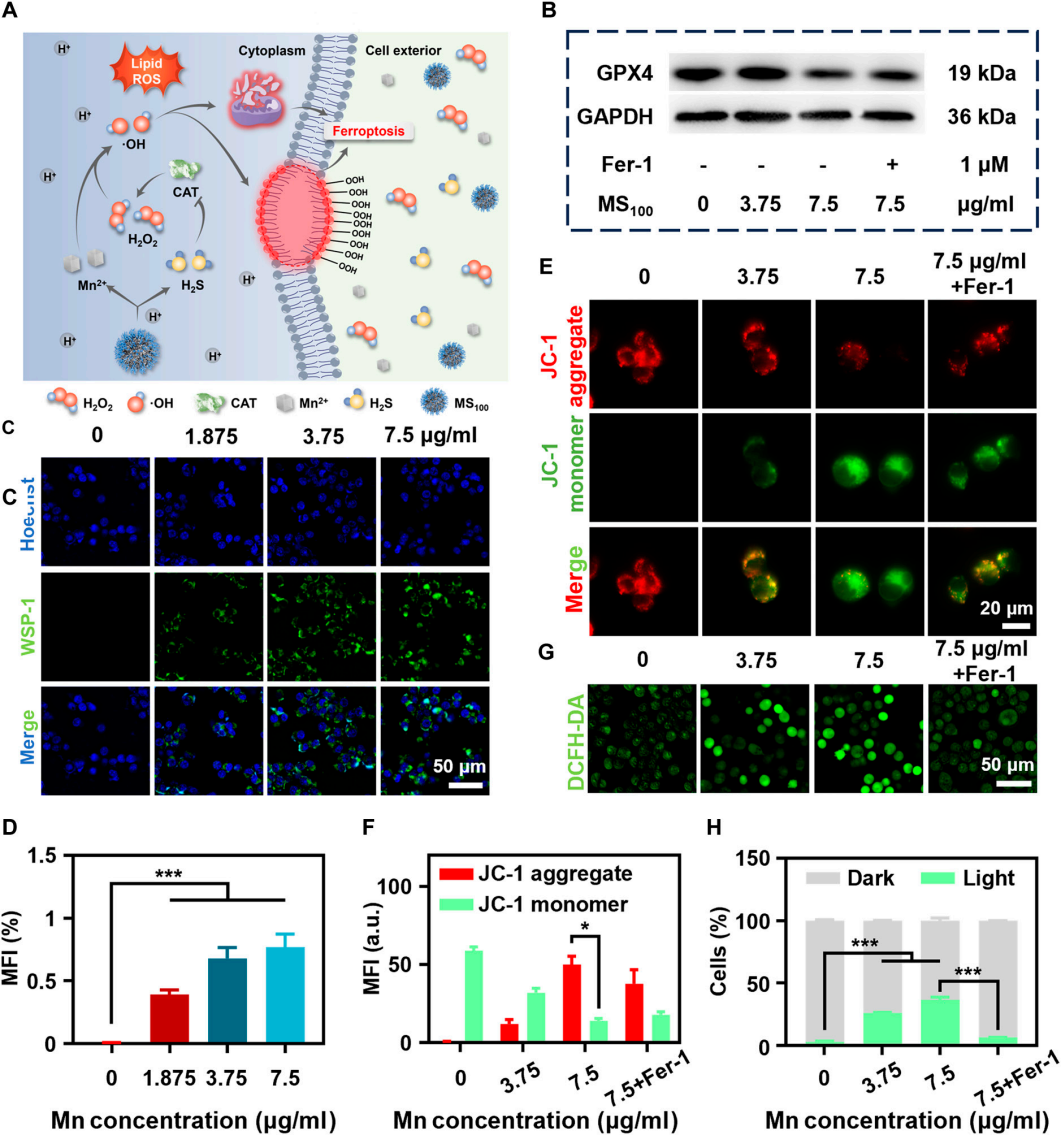
Synergistic therapeutic mechanism of MS_100_. (A) Schematic diagram of CDT–gas synergistic therapeutic mechanism. (B) Protein expression of GPX4. (C) Fluorescence images of H_2_S production and (D) the corresponding quantitative analysis. Green, WSP-1 represented H_2_S. (E) Fluorescence images of LLC cells treated with JC-1 probe and (F) the corresponding quantitative analysis. (G) Fluorescence images of LLC cells treated with DCFH-DA probe and (H) the corresponding quantitative analysis. Data are represented as mean ± SD and analyzed by one-way ANOVA. *P < 0.05, ***P < 0.001 (*n* = 3 independent experiments).

MS_100_ can decompose into Mn^2+^ and H_2_S under acidic conditions, achieving chemodynamic therapy (CDT)–gas synergistic therapeutic effects on tumor cells through cascade ferroptosis. Through the Fenton reaction, Mn^2+^ produced by decomposition transforms the overexpressed H_2_O_2_ within tumor cells into cytotoxic •OH, facilitating LPO in cellular and organelle membranes, ultimately inducing ferroptosis in tumor cells. Simultaneously, H₂S further increases intracellular H₂O₂ levels by inhibiting CAT activity, providing a continuous substrate for the Fenton reaction, thereby enabling cascade ferroptosis in tumor cells (Fig. 7A). This mechanistic pathway was further delineated by Western blot analysis, which revealed alterations in the protein levels of GPX4 following MS_100_ treatment (Fig. 7B and Fig. S11). Notably, the administration of Fer-1, a ferroptosis inhibitor, partially restored GPX4 levels, confirming that MS_100_ induces ferroptosis-mediated tumor cell death.

Further validation of H_2_S production after MS_100_ co-incubation in LLC cells was achieved using the WSP-1 probe. A progressive elevation in H_2_S levels was noted by the WSP-1 probe when LLC cells were exposed to MS_100_, showing a dependency on the concentration of the compound (Fig. 7C and D). As shown in Fig. 7E and F, the mitochondrial membrane potential decreased with increasing MS_100_ concentration, causing JC-1 to transition from an aggregated state (red) to a dispersed state (green), and Fer-1 was able to reverse this phenomenon. When LLC cells were treated with MS_100_, a dose-dependent increase in fluorescence signal was observed using DCFH-DA as an •OH monitoring probe, confirming the effective generation of •OH in tumor cells. Fer-1 partially inhibited the production of •OH (Fig. 7G and H). Additionally, CAT activity assays after MS_100_ treatment underscored the inhibition of CAT (Fig. S12). TEM images of MS_100_-treated LLC cells revealed critical morphological changes in the mitochondria, including rounding, loss of cristae, rupture of the outer membrane, and vesiculation (black arrows) (Fig. S13), providing definitive evidence of MS_100_’s capability to induce ferroptosis in tumor cells.

### In vivo antitumor efficacy and safety profile of MS_100_

**Fig. 8.**
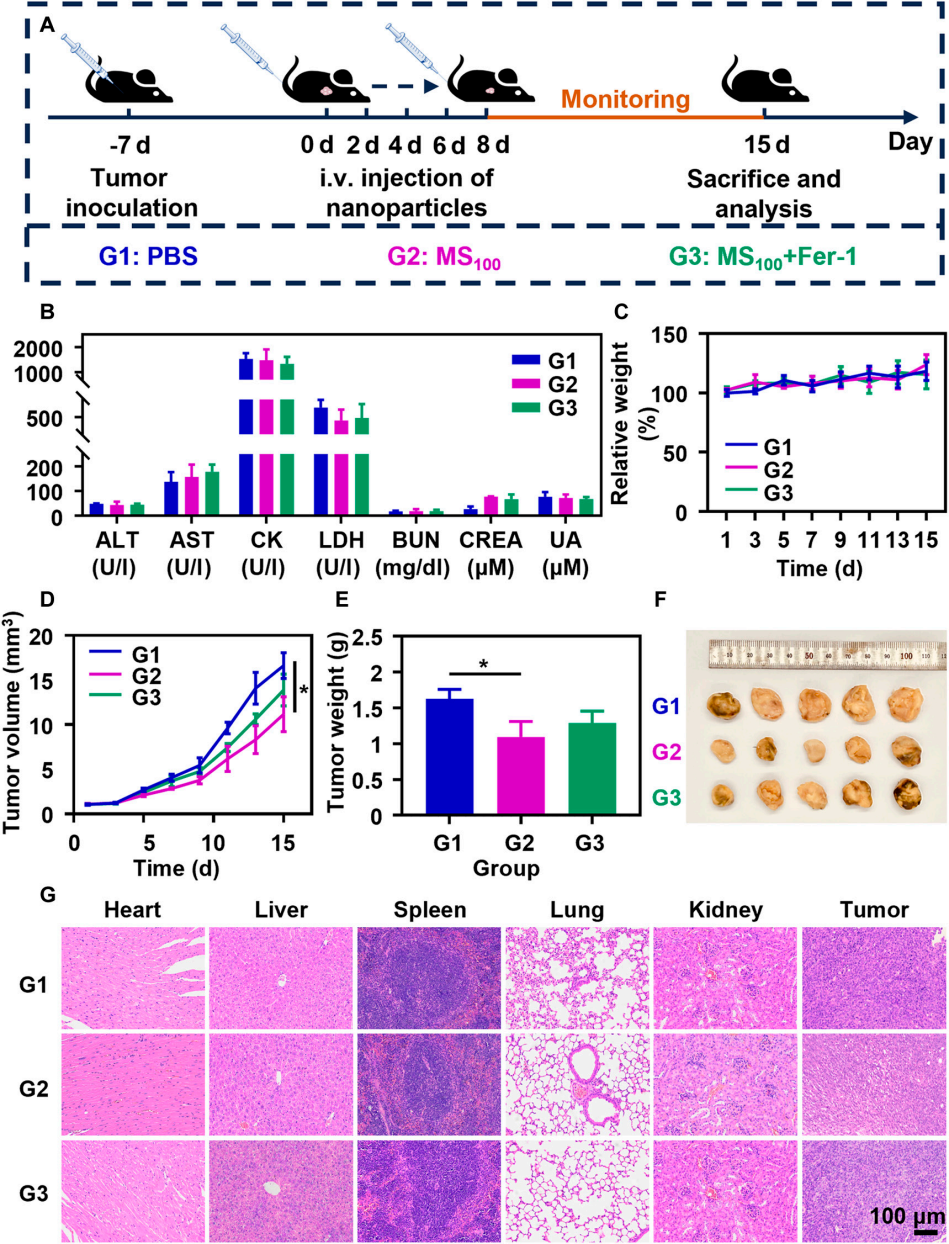
In vivo antitumor efficacy of MS_100_. (A) Schematic of the mice treatment protocol. (B to D) Changes in serological markers, mouse body weight, and tumor volume. (E and F) Weight and photographs of ex vivo tumors. (G) Representative pathological H&E staining images of major organs and tumors from different treatment groups. Data are represented as mean ± SD and analyzed by one-way ANOVA. *P < 0.05 (*n* = 3 independent experiments).

Considering that MS_100_ has already demonstrated superior anticancer effects in vitro, we next explored its in vivo therapeutic potential. The tumor treatment protocol is depicted in Fig. 8A. Mice were randomly assigned to 1 of 3 groups: PBS (G1), MS_100_ (G2), and MS_100_ + Fer-1 (G3). Mice received intravenous injections of 2 mg (Mn)/kg (mouse body weight) of MS_100_ via the tail vein every other day, with tumor volumes and body weights measured every 2 days. The results indicated no significant weight changes across all groups during the treatment period (Fig. 8C), and all biochemical parameters remained within normal ranges (Fig. 8B). Tumor growth was partially inhibited after the administration of MS_100_. The addition of Fer-1 partially reversed the tumoricidal effects, leading to an increase in tumor volume (Fig. 8D). The weight of ex vivo tumors at the treatment endpoint reflected a similar trend (Fig. 8E and F). Excised tumors were subjected to H&E staining (Fig. 8G). The results revealed that in the PBS group, tumor cells exhibited rich chromatin, darker staining, larger nuclei, and more frequent mitotic figures. In contrast, tumor cells treated with MS_100_ displayed chromatin condensation and vacuolization. Cells in the MS_100_ + Fer-1 group showed intermediate characteristics. These findings indicate that MS_100_ exhibits excellent biocompatibility and low toxicity while also demonstrating significant in vivo antitumor effects.

## Discussion

MRI is widely utilized in medical diagnostics due to its noninvasive nature and high-resolution capabilities. This study highlights the potential of γ-MnS NPs as multifunctional contrast agents, offering enhanced T_1_ contrast and therapeutic benefits, positioning them as a promising alternative to gadolinium-based agents. Our synthesis of spherical γ-MnS NPs revealed that solvent ratios (DEG/EG) significantly influence particle size. Higher DEG content resulted in smaller nanospheres, attributed to (a) the ether group in DEG reducing the molecular polarity and solubility of Mn acetylacetonate, leading to smaller, more dispersed Mn cores; (b) lower solubility inhibiting redissolution and limiting nanosphere growth; and (c) DEG’s longer chains and increased viscosity affecting particle formation.

In vitro and in vivo studies have demonstrated that particle size significantly affects cellular uptake and retention at tumor sites. γ-MnS NPs decompose in acidic tumor microenvironments, releasing Mn^2+^ and hydrogen sulfide (H₂S). Mn^2+^ enhances MRI contrast, while H₂S inhibits CAT activity, promoting H₂O₂ accumulation and enhancing ROS-mediated tumor cell apoptosis. Smaller particles exhibit stronger acid responsiveness due to their larger surface area, increasing interactions with protons and leading to more efficient Mn^2+^ release. These findings suggest that the size-dependent acid responsiveness of γ-MnS enhances both imaging and therapeutic potential in the tumor microenvironment, positioning it as a viable candidate for theranostic applications.

γ-MnS NPs show significant potential as a theranostic platform, combining diagnostic and therapeutic functions. Their rough surface allows for drug loading, enabling targeted drug delivery alongside imaging capabilities. Future research should focus on enhancing tumor-specific targeting while minimizing systemic exposure. Although short-term biocompatibility is promising, long-term studies on biodistribution, clearance, and potential accumulation are necessary, given the role of Mn^2+^ in biological systems. Additionally, exploring the potential of γ-MnS NPs as carriers for other therapeutic agents could further extend their functionality, providing a comprehensive solution for personalized, low-toxicity, and effective cancer treatment.

## Data Availability

Data will be made available on request.

## References

[1] Wahsner J, Gale EM, Rodríguez-Rodríguez A, Caravan P. Chemistry of MRI contrast agents: Current challenges and new frontiers. Chem Rev. 2019;119(2):957–1057. 30350585 10.1021/acs.chemrev.8b00363 PMC6516866

[2] Liu H, Lu C, Han L, Zhang X, Song G. Optical-magnetic probe for evaluating cancer therapy. Coord Chem Rev. 2021;441: Article 213978.

[3] Brito B, Price TW, Gallo J, Bañobre-López M, Stasiuk GJ. Smart magnetic resonance imaging-based theranostics for cancer. Theranostics. 2021;11(18):8706–8737. 34522208 10.7150/thno.57004 PMC8419031

[4] Zhang G, Du R, Qian J, Zheng X, Tian X, Cai D, He J, Wu Y, Huang W, Wang Y. A tailored nanosheet decorated with a metallized dendrimer for angiography and magnetic resonance imaging-guided combined chemotherapy. Nanoscale. 2018;10(1):488–498. 10.1039/c7nr07957e 29231948

[5] Liu S, Jiang Y, Liu P, Yi Y, Hou D, Li Y, Liang X, Wang Y, Li Z, He J, et al. Single-atom gadolinium nano-contrast agents with high stability for tumor T_1_ magnetic resonance imaging. ACS Nano. 2023;17(9):8053–8063. 37092888 10.1021/acsnano.2c09664

[6] Pinter NK, Klein JP, Mechtler LL. Potential safety issues related to the use of gadolinium-based contrast agents. Continuum. 2016;22(5, Neuroimaging):1678–1684. 27740994 10.1212/CON.0000000000000378

[7] Sieber MA, Lengsfeld P, Frenzel T, Golfier S, Schmitt-Willich H, Siegmund F, Walter J, Weinmann HJ, Pietsch H. Preclinical investigation to compare different gadolinium-based contrast agents regarding their propensity to release gadolinium in vivo and to trigger nephrogenic systemic fibrosis-like lesions. Eur Radiol. 2008;18(10):2164–2173. 18545998 10.1007/s00330-008-0977-y

[8] Puttagunta NR, Gibby WA, Smith GT. Human in vivo comparative study of zinc and copper transmetallation after administration of magnetic resonance imaging contrast agents. Investig Radiol. 1996;31(12):739–742. 8970874 10.1097/00004424-199612000-00001

[9] He T, Qin X, Jiang C, Jiang D, Lei S, Lin J, Zhu W-G, Qu J, Huang P. Tumor pH-responsive metastable-phase manganese sulfide nanotheranostics for traceable hydrogen sulfide gas therapy primed chemodynamic therapy. Theranostics. 2020;10(6):2453–2462. 32194812 10.7150/thno.42981 PMC7052883

[10] Liu H, Mu M, Hou Y, Gong Y, Wang C, Ma G, Guo K, Ma L, Sun X. A novel CRISPR/Cas9-encapsulated biomimetic manganese sulfide nanourchins for targeted magnetic resonance contrast enhancement and self-enhanced chemodynamics-gene-immune synergistic tumor therapy. Adv Funct Mater. 2024;34(36):2401370.

[11] Ma G, Zhang X, Zhao K, Zhang S, Ren K, Mu M, Wang C, Wang X, Liu H, Dong J, et al. Polydopamine nanostructure-enhanced water interaction with pH-responsive manganese sulfide nanoclusters for tumor magnetic resonance contrast enhancement and synergistic ferroptosis–photothermal therapy. ACS Nano. 2024;18(4):3369–3381. 38251846 10.1021/acsnano.3c10249

[12] Zhang L, Yang Z, Ren J, Ba L, He W, Wong C-Y. Multifunctional oxygen-enriching nano-theranostics for cancer-specific magnetic resonance imaging and enhanced photodynamic/photothermal therapy. Nano Res. 2020;13(5):1389–1398.

[13] Mauri M, Collico V, Morelli L, Das P, Garcia I, Penaranda Avila J, Bellini M, Rotem R, Truffi M, Corsi F. MnO nanoparticles embedded in functional polymers as T_1_ contrast agents for magnetic resonance imaging. ACS Appl Nano Mater. 2020;3(4):3787–3797.

[14] Guo B, Zhao J, Zhang Z, An X, Huang M, Wang S. Intelligent nanoenzyme for T_1_-weighted MRI guided theranostic applications. Chem Eng J. 2020;391: Article 123609.

[15] Sun X, Zhang G, Du R, Xu R, Zhu D, Qian J, Bai G, Yang C, Zhang Z, Zhang X. A biodegradable MnSiO3@ Fe3O4 nanoplatform for dual-mode magnetic resonance imaging guided combinatorial cancer therapy. Biomaterials. 2019;194:151–160. 30594744 10.1016/j.biomaterials.2018.12.004

[16] Zhu X, Xiong H, Zhou Q, Zhao Z, Zhang Y, Li Y, Wang S, Shi S. A pH-activatable MnCO3 nanoparticle for improved magnetic resonance imaging of tumor malignancy and metastasis. ACS Appl Mater Interfaces. 2021;13(16):18462–18471. 33871955 10.1021/acsami.0c22624

[17] Lee KK, Lee J-H, Lee SC, Lee C-S. MnCO3-mineralized polydopamine nanoparticles as an activatable theranostic agent for dual-modality imaging-guided photothermal therapy of cancers. Theranostics. 2022;12(15):6762–6778. 36185599 10.7150/thno.77060 PMC9516237

[18] Miao Y, Xie Q, Zhang H, Cai J, Liu X, Jiao J, Hu S, Ghosal A, Yang Y, Fan H. Composition-tunable ultrasmall manganese ferrite nanoparticles: Insights into their in vivo T_1_ contrast efficacy. Theranostics. 2019;9(6):1764–1776. 31037137 10.7150/thno.31233 PMC6485191

[19] Xiao S, Yu X, Zhang L, Zhang Y, Fan W, Sun T, Zhou C, Liu Y, Liu Y, Gong M, et al. Synthesis of PEG-coated, ultrasmall, manganese-doped iron oxide nanoparticles with high relaxivity for T_1_/ T_2_ dual-contrast magnetic resonance imaging. Int J Nanomedicine. 2019;14:8499–8507. 31695377 10.2147/IJN.S219749 PMC6817351

[20] Díez-Villares S, Ramos-Docampo MA, da Silva-Candal A, Hervella P, Vázquez-Ríos AJ, Dávila-Ibáñez AB, López-López R, Iglesias-Rey R, Salgueiriño V, Mdl F. Manganese ferrite nanoparticles encapsulated into vitamin E/sphingomyelin nanoemulsions as contrast agents for high-sensitive magnetic resonance imaging. Adv Funct Mater. 2021;10(21):2101019. 10.1002/adhm.202101019 PMC11469163 34415115

[21] Hou X, Yang X, Xu Y, Lin J, Zhang F, Duan X, Liu S, Liu J, Shen J, Shuai X, et al. Manganese-doped mesoporous polydopamine nanoagent for T_1_–T_2_ magnetic resonance imaging and tumor therapy. Nano Res. 2023;16(2):2991–3003.

[22] Jiang Z, Yuan B, Qiu N, Wang Y, Sun L, Wei Z, Li Y, Zheng J, Jin Y, Li Y, et al. Manganese-zeolitic imidazolate frameworks-90 with high blood circulation stability for MRI-guided tumor therapy. Nano Micro Lett. 2019;11(1):61. 10.1007/s40820-019-0292-y PMC7770799 34138009

[23] Yang C, Song G, Yuan H, Yang Y, Wang Y, Ye D, Meng H, Huan S, Zhang X-B. Manganese–fluorouracil metallodrug nanotheranostic for MRI-correlated drug release and enhanced chemoradiotherapy. CCS Chem. 2021;3(4):1116–1128.

[24] Corpas FJ, Barroso JB, González-Gordo S, Muñoz-Vargas MA, Palma JM. Hydrogen sulfide: A novel component in Arabidopsis peroxisomes which triggers catalase inhibition. J Integr Plant Biol. 2019;61(7):871–883. 30652411 10.1111/jipb.12779

[25] Fang C, Cen D, Wang Y, Wu Y, Cai X, Li X, Han G. ZnS@ZIF-8 core-shell nanoparticles incorporated with ICG and TPZ to enable H(2)S-amplified synergistic therapy. Theranostics. 2020;10(17):7671–7682. 32685012 10.7150/thno.45079 PMC7359076

[26] Elumalai K, Srinivasan S, Shanmugam A. Review of the efficacy of nanoparticle-based drug delivery systems for cancer treatment. Biomed Tech. 2024;5:109–122.

[27] Xiao J, Zhang G, Qian J, Sun X, Tian J, Zhong K, Cai D, Wu Z. Fabricating high-performance T 2-weighted contrast agents *via* adjusting composition and size of nanomagnetic iron oxide. ACS Appl Mater Interfaces. 2018;10(8):7003–7011. 29392939 10.1021/acsami.8b00428

[28] Gan Y, Zhang J, Lei S, Yan M, Xie W, Qi X, Wang H, Xiao J, Chen S, Li S, et al. Atomically precise multi-domain GdxFe3− xO4 nanoclusters with modulated contrast properties for T_2_-weighted magnetic resonance imaging of early orthotopic cancer. Chem Eng J. 2022;429: Article 132255.

[29] Zhou Z, Huang D, Bao J, Chen Q, Liu G, Chen Z, Chen X, Gao J. A synergistically enhanced T(1) -T(2) dual-modal contrast agent. Adv Mater. 2012;24(46):6223–6228. 22972529 10.1002/adma.201203169 PMC3634350

[30] Sreekumar S, Goycoolea FM, Moerschbacher BM, Rivera-Rodriguez GR. Parameters influencing the size of chitosan-TPP nano- and microparticles. Sci Rep. 2018;8(1):4695. 29549295 10.1038/s41598-018-23064-4 PMC5856823

[31] Takeshita S, Honda J, Isobe T, Sawayama T, Niikura S. Size-tunable solvothermal synthesis of Zn2GeO4: Mn^2+^ nanophosphor in water/diethylene glycol system. Cryst Growth Des. 2010;10(10):4494–4500.

[32] Zhao Y, Zhang Z, Pan Z, Liu Y. Advanced bioactive nanomaterials for biomedical applications. Exploration. 2021;1(3):20210089. 37323697 10.1002/EXP.20210089 PMC10191050

[33] Cheng J, Zhu Y, Dai Y, Li L, Zhang M, Jin D, Liu M, Yu J, Yu W, Su D, et al. Gas-mediated tumor energy remodeling for sensitizing mild photothermal therapy. Angew Chem. 2023;62(27): Article e202304312. 37137872 10.1002/anie.202304312

[34] Li Y, Lin J, He Y, Wang K, Huang C, Zhang R, Liu X. Tumour-microenvironment-responsive Na2S2O8 nanocrystals encapsulated in hollow organosilica–metal–phenolic networks for cycling persistent tumour-dynamic therapy. Exploration. 2024;4(2):20230054. 38855614 10.1002/EXP.20230054 PMC11022624

[35] Gao Z, Zheng S, Kamei K-i, Tian C. Recent progress in cancer therapy based on the combination of ferroptosis with photodynamic therapy. Acta Mater Med. 2022;1(4):411–426.

[36] Di G, Zhu Z, Dai Q, Zhang H, Shen X, Qiu Y, Huang Y, Yu J, Yin D, Küppers S. Wavelength-dependent effects of carbon quantum dots on the photocatalytic activity of g-C_3_N_4_ enabled by LEDs. Chem Eng J. 2020;379: Article 122296.

