# Supplementary material for: Customized Sized Manganese Sulfide Nanospheres as Efficient T1 MRI Contrast Agents for Enhanced Tumor Theranostics
Source: Biomater Res. 2024 Dec 11;28:0116. doi: 10.34133/bmr.0116 (PMC11632153; doi:10.34133/bmr.0116)
Supplement: Supplementary 1 — Figs. S1 to S13 Table S1 [file bmr.0116.f1.docx]

**Supporting Information**

Customized Sized Manganese Sulfide Nanospheres as Efficient T1 MRI Contrast Agents for Enhanced Tumor Theranostics

Yufang Gong^a,#^, Kai Guo^b,#^, Siyu Cai^b^, Ke Ren^b^, Liya Tian^b^, Yingqi Wang^b^, Mengyao Mu^b^, Qingwei Meng^a,*^, Jie Liu^b,*^, Xiao Sun^b,*^

^a^Department of Medical Oncology, Harbin Medical University Cancer Hospital, Harbin, 150081, Heilongjiang, China

^b^Medical Science and Technology Innovation Center, Shandong First Medical University & Shandong Academy of Medical Sciences, Jinan 250000, China

*Corresponding authors: mengqw@hrbmu.edu.cn (Q. Meng); linchuangliujie@163.com (J. Liu); sunxiao@sdfmu.edu.cn (X. Sun)

1. **Supporting Figures**


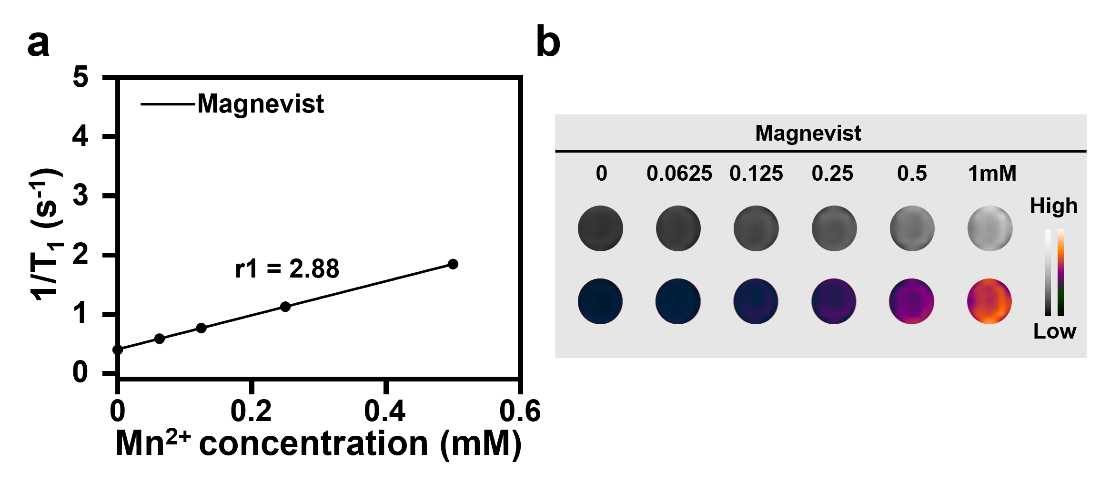


**Figure S1.** a) T_1_ relaxation rates of Magnevist in the same acidic environment from a 0.5 T MRI system (pH=5.5) in vitro. b) T1-weighted MR image of Magnevist from a 3.0 T MRI system.


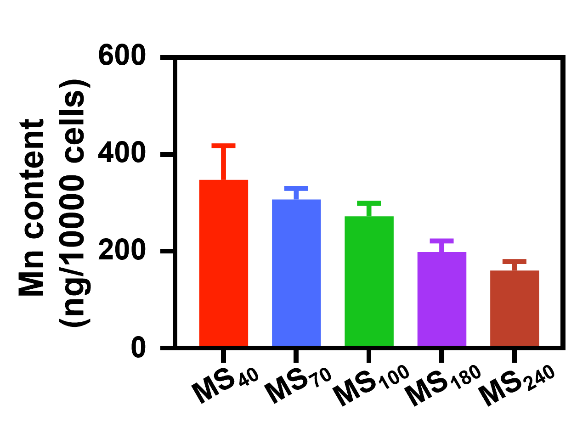


**Figure S2.** ICP-OES quantification of MnS@PAA in LLC cells (n = 3).


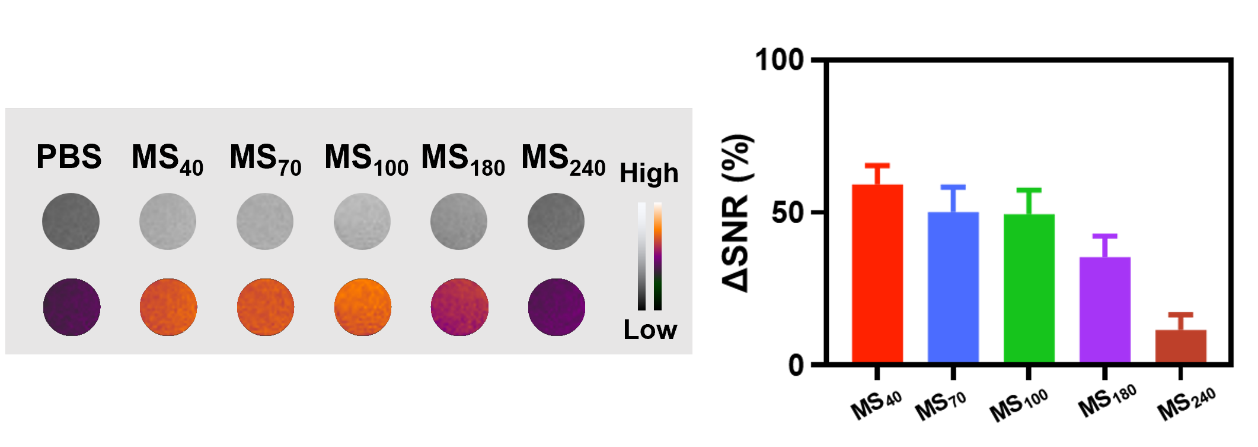


**Figure S3.** T1 MR performance of MnS@PAA with different sizes in hydrogel (9.4T) and its qualification.


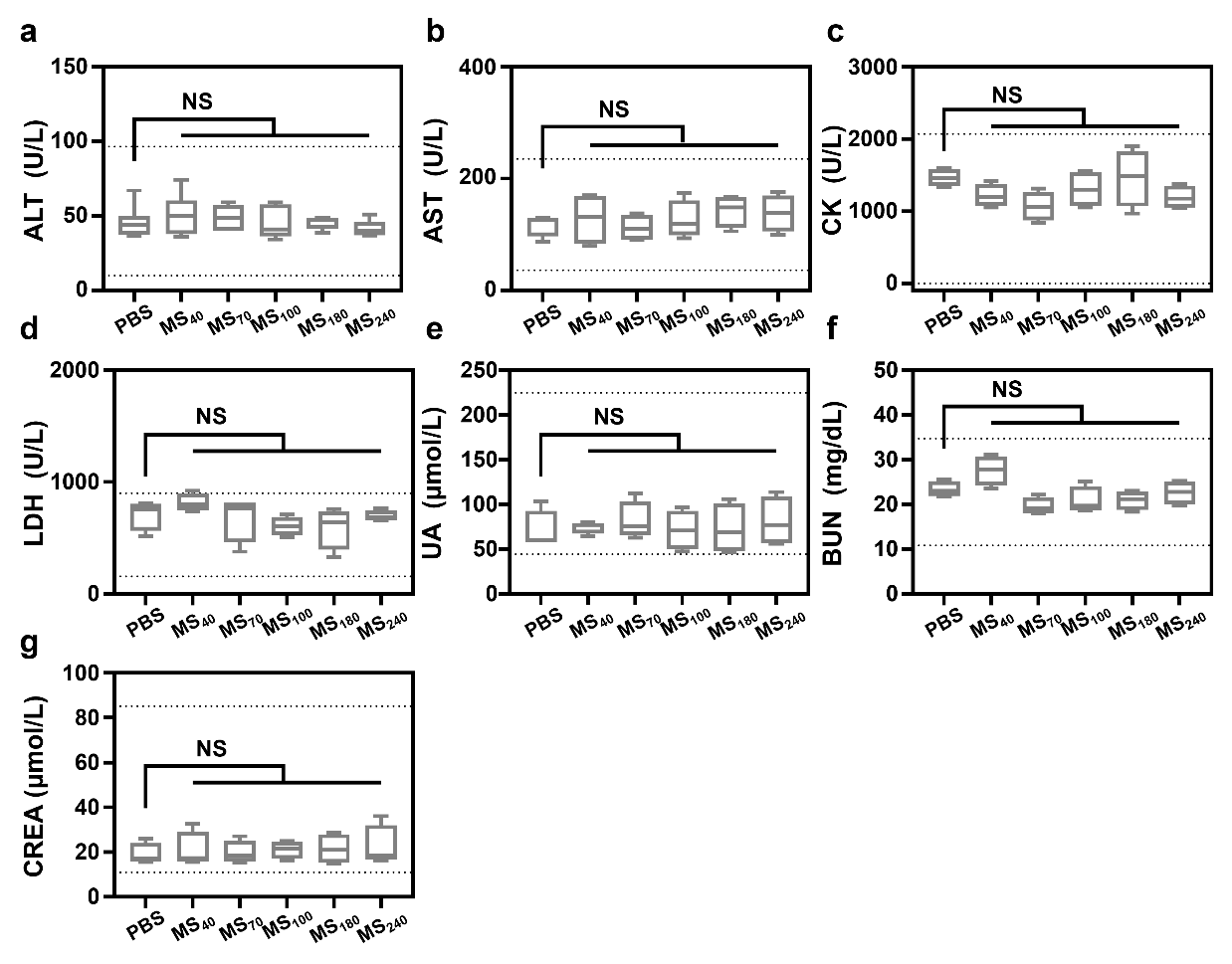


**Figure S4.** Blood biochemical indices of mice treated with MnS@PAA (n = 4). Data represented mean ± standard deviation and analysis by one-way ANOVA.


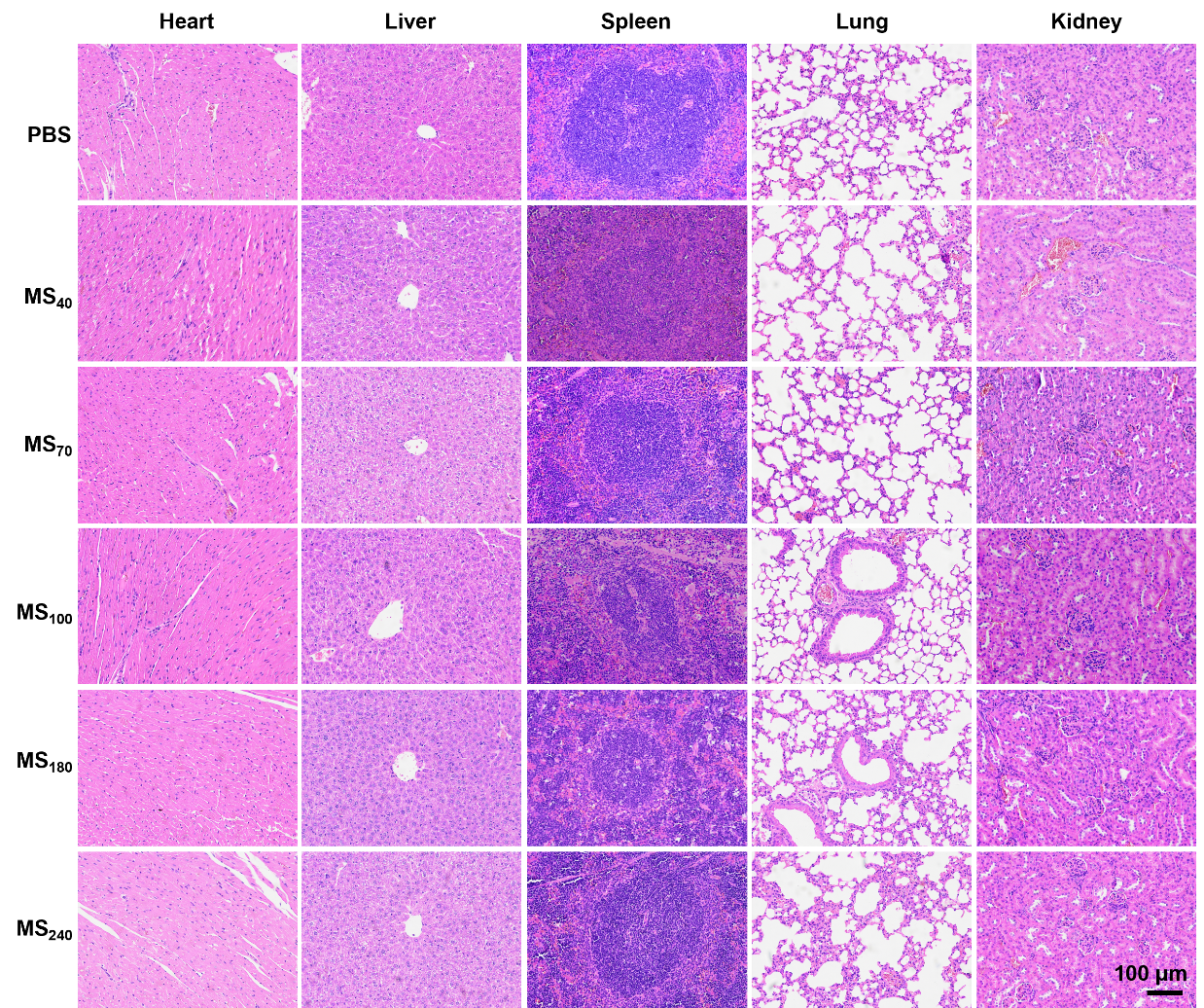


**Figure S5.** H&E staining of the major organs after different treatments.


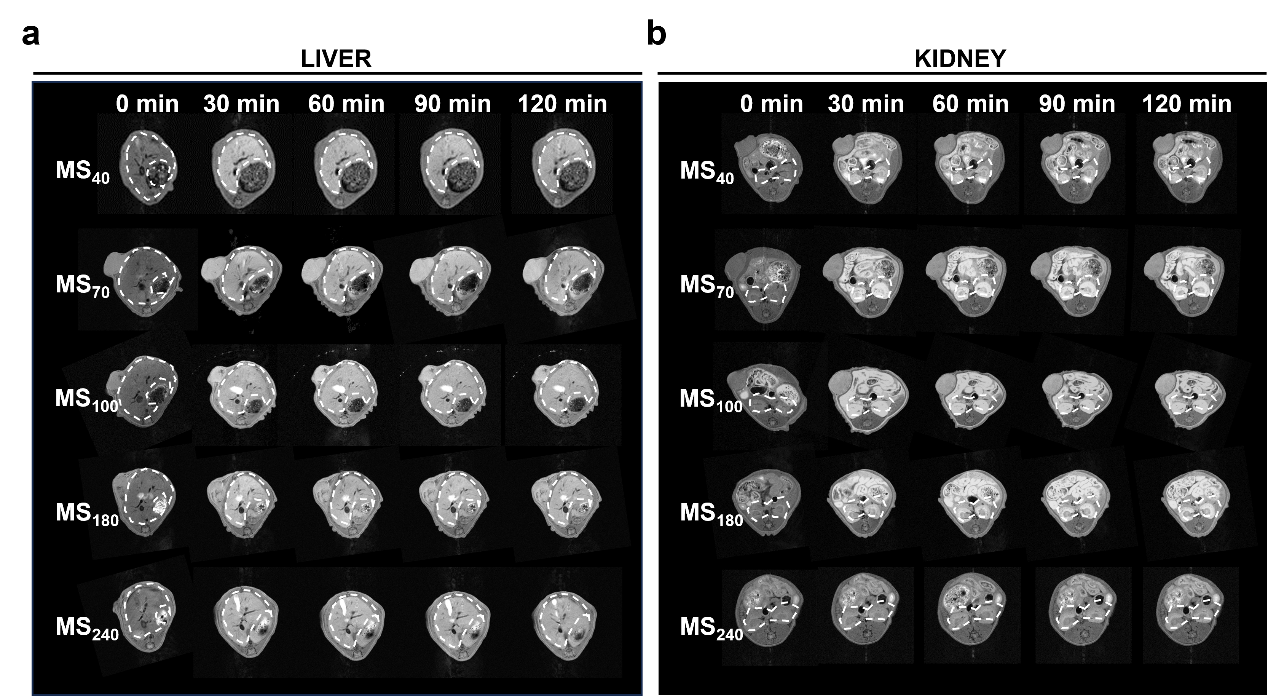


**Figure S6.** T1-weighted images of mouse liver and kidneys of MnS@PAA with different sizes.


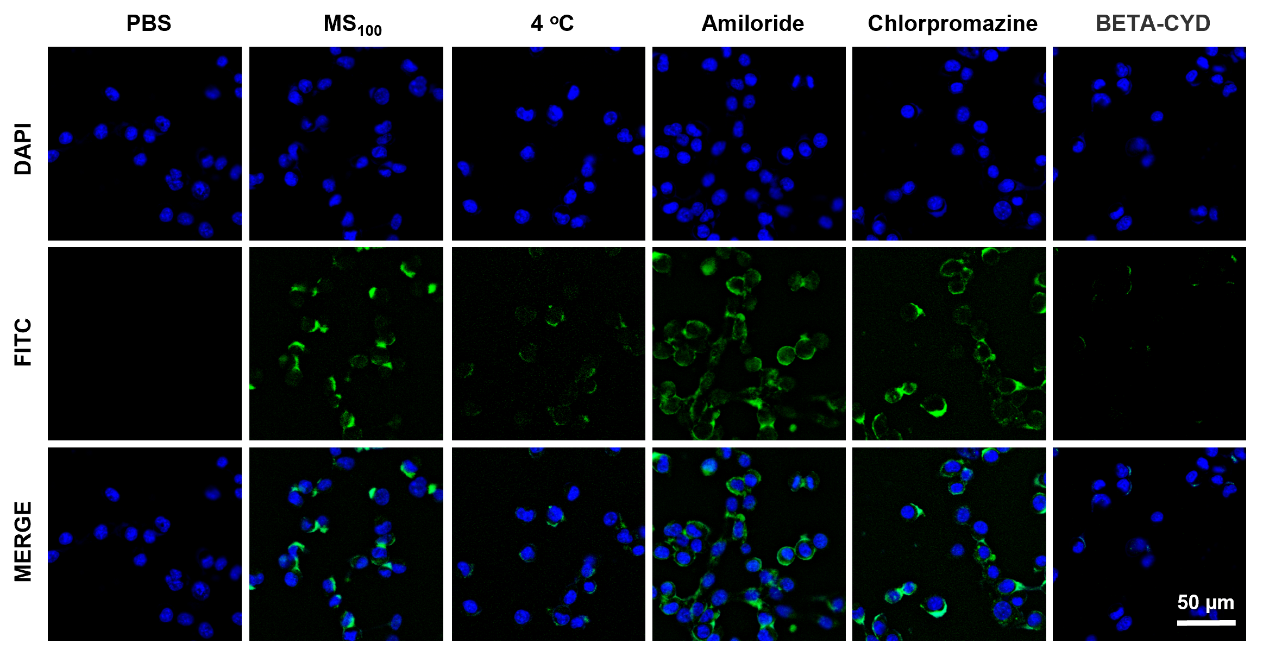


**Figure S****7.** MS_100_ uptake in the presence of different inhibitors or 4^o^C.


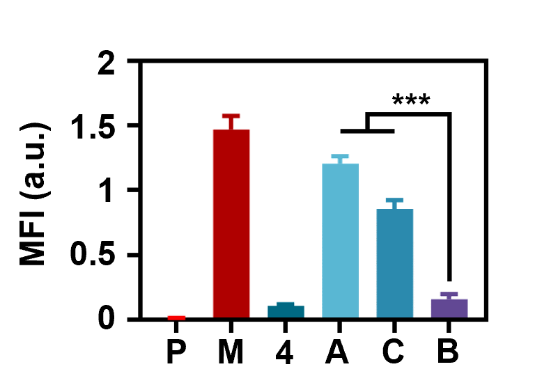


**Figure S8.** Qualification of MS_100_ uptake after treatment with various inhibitors or 4 ^o^C. (P, PBS. M, MS_100_. 4, 4 ^o^C. A, Amiloride. C, Chlorpromazine. B, BETA-CYD. n = 3). Data represented mean ± standard deviation and analysis by one-way ANOVA. ***p < 0.001,

**
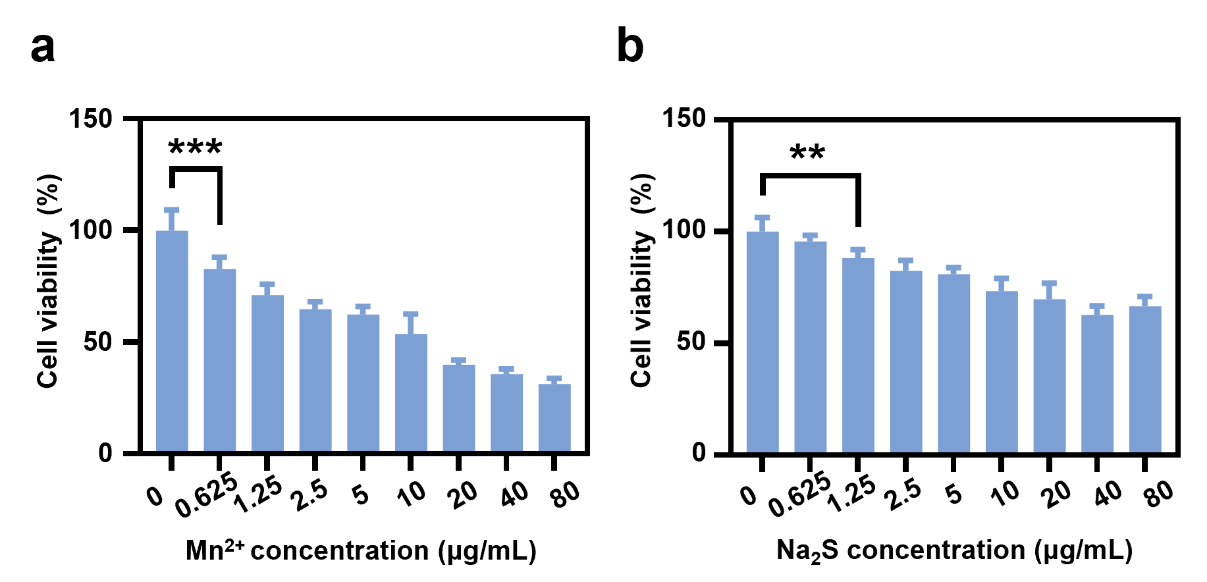
**

**Figure S9.** Cell viability of LLC under different concentrations of (a) MnCl_2_ and (b) Na_2_S for 24 h (n = 4). Data represented mean ± standard deviation and analysis by one-way ANOVA. **p < 0.01, ***p < 0.001.

**
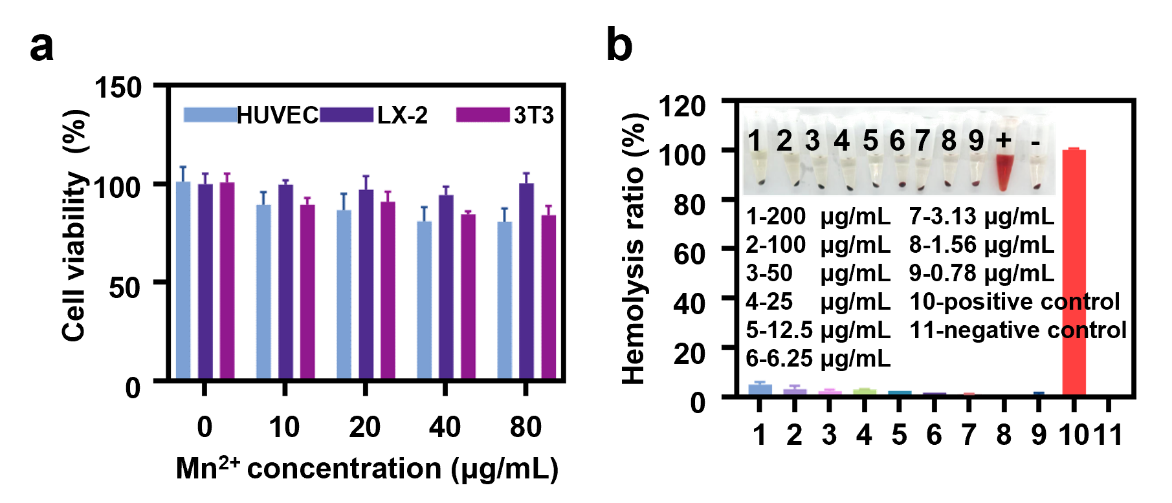
**

**Figure S10.** (a) Cell viability of HUVEC, LX-2 and NIH/3T3 cells treated by MS_100_ for 24 h. (b) Photograph of hemolysis test and hemolysis rate. n=3.


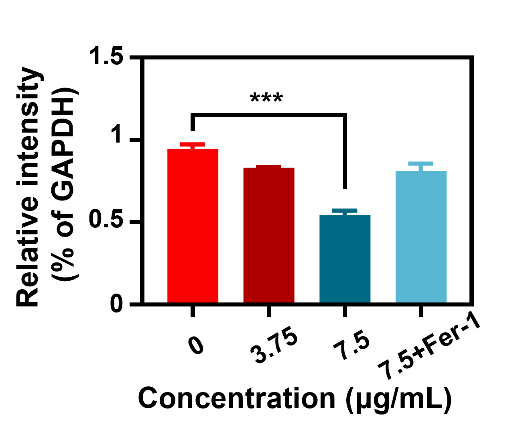


**Figure S11.** Quantification of GPX4 protein expression in different treatment groups (n = 3). Data represented mean ± standard deviation and analysis by one-way ANOVA. ***p < 0.001.

**
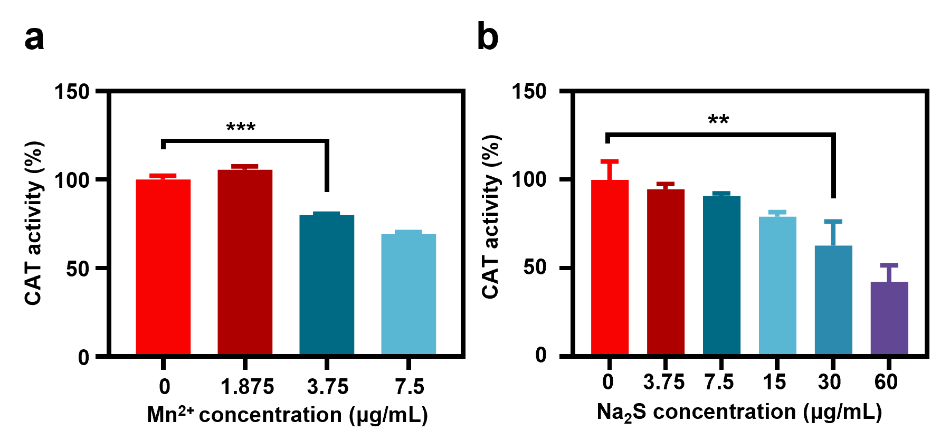
**

**Figure S12.** Detection results of intracellular CAT enzyme activity after the addition of (a) MS_100_ and (b) hydrogen sulfide donor (n = 3). Data represented mean ± standard deviation and analysis by one-way ANOVA. **p < 0.01, ***p < 0.001.


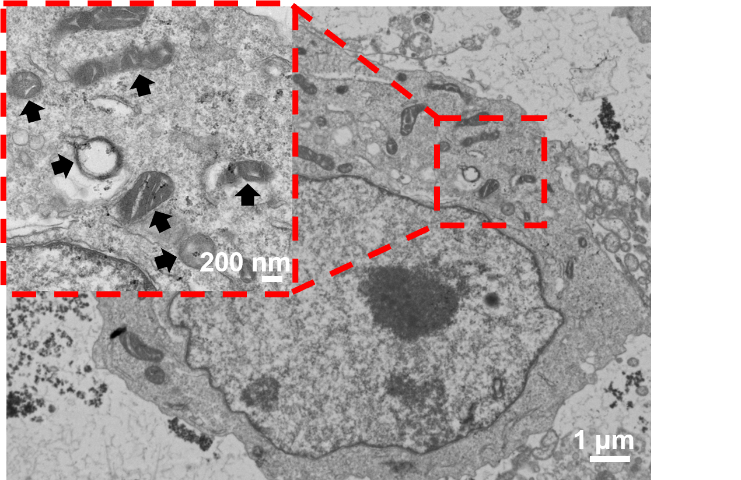


**Figure S13.** Bio-TEM images of LLC cells treated with MS_100_.

**Table S1. Comparison of Mn2+ release from five different sizes of MnS@PAA at varying pH levels.**

| Sample | Mn content  in pH 7.4 (%) | Mn content  in pH 5.5 (%) |
| --- | --- | --- |
| MS_40_ | 29.66 | 66.34 |
| MS_70_ | 33.66 | 60.71 |
| MS_100_ | 28.32 | 54.3 |
| MS_180_ | 28 | 44.9 |
| MS_240_ | 23.56 | 40.61 |
